# Selective HDAC6 inhibitor WT161 modulates the VLA-4/FAK pathway by inhibiting PKA activity in acute lymphoblastic leukemia

**DOI:** 10.1038/s41598-025-23887-y

**Published:** 2025-11-17

**Authors:** Chengfang Lv, Yingling Zhao, Ling Luo, Chuntao Ye, Chen Rao, Bishan Feng, Wenqing Yu, Xiaoling Xiao, Cuiping Wang, Wangxiang Huang

**Affiliations:** Department of Hematology, Shenzhen Longgang Central Hospital, Shenzhen, 518100 China

**Keywords:** WT161, Very late antigen-4, Focal adhesion kinase, Acute lymphoblastic leukemia, PKA, Haematological cancer, Leukaemia

## Abstract

Acute lymphoblastic leukemia (ALL) is a genetically heterogeneous malignancy often associated with poor prognosis due to chemotherapy resistance and relapse. Histone deacetylase (HDAC) inhibitors represent an emerging class of epigenetic antitumor drugs. Among them, HDAC6, which is an enzyme that deacetylates α-tubulin, is increasingly recognized as a potential therapeutic target in hematologic malignancies. The adhesion molecule very late antigen 4 (VLA-4) plays a key role in cell adhesion-mediated drug resistance (CAMDR). We hypothesized that inhibiting HDAC6 could counteract CAMDR mediated by crosstalk between α-tubulin and integrin signaling. This study therefore investigated the therapeutic potential of WT161, a selective HDAC6 inhibitor, focusing on its role in targeting of the VLA-4/Focal adhesion kinase (FAK) signaling pathway in ALL. Human B-ALL (BALL-1, NALM6) and T-ALL (Jurkat, MOLT-4) cell lines were treated with WT161, and its effects on proliferation, adhesion, migration, apoptosis, and cell cycle progression were evaluated. Protein expression and phosphorylation were analyzed by Western blot, and VLA-4 expression was assessed using immunofluorescence. In vivo, NOD/SCID mice xenografted with ALL cells were treated with WT161, vincristine, or their combination. WT161 significantly inhibited proliferation, reduced adhesion and migration, and induced apoptosis in ALL cells. Mechanistically, it decreased intracellular cAMP levels, thereby inhibiting Protein kinase A (PKA) activity and suppressing the FAK signaling pathway. In xenograft models, WT161 exhibited anti-tumor effects, which were enhanced in combination with vincristine. This study highlights WT161’s therapeutic potential in ALL, demonstrating its ability to inhibit PKA activity and disrupt the VLA-4/FAK pathway, thereby offering a promising novel strategy for ALL treatment.

**Supplementary Information:**

The online version contains supplementary material available at 10.1038/s41598-025-23887-y.

## Introduction

Acute lymphoblastic leukemia (ALL) is a genetically heterogeneous malignancy characterized by the accumulation of immature lymphocytes in the bone marrow and peripheral blood^1^. Despite considerable therapeutic advances, many pediatric and most adult patients experience relapse or suffer from treatment-related complications, resulting in poor long-term survival^2,3^. A comprehensive understanding of the molecular mechanisms driving ALL pathogenesis is therefore essential for improving clinical outcomes and identifying novel therapeutic targets.

Histone acetylation plays a crucial role in ALL by regulating gene expression, cell cycle control, apoptosis, and differentiation. Histone deacetylases (HDACs) are key enzymes that modulate these processes by removing acetyl groups from both histone and non-histone proteins^4^. Although HDAC inhibitors have demonstrated therapeutic potential in various malignancies, their clinical application is often limited by offtarget cytotoxicity and adverse effects^5,6^. In this context, selective inhibition of HDAC6, a cytoplasmic protein with diverse substrates such as heat shock protein 90 (HSP90), and PTEN, offers a promising targeted strategy^7–10^. WT161, a selective HDAC6 inhibitor, has shown significant antitumor efficacy in multiple myeloma, retinoblastoma, and breast cancer^11–13^. However, its effects on ALL remains largely unexplored.

Very late antigen-4 (VLA-4, integrin α4β1), a key leukocyte integrin composed of CD49d (α4) and CD29 (β1) subunits, mediates the adhesion of ALL cells to the vascular endothelium and extracellular matrix^14^. This interaction facilitates leukemic cell migration and contributes to disease progression. Elevated VLA-4 expression correlates with poor prognosis in relapsed ALL^15^. Focal adhesion kinase (FAK), a central mediator of integrin signaling, is critical for cell adhesion and migration^16^. Protein kinase A (PKA) activates VLA-4 signaling by phosphorylating its α4 subunit at Ser988^17^. Additionally, HDAC6 inhibitors have been shown to decrease intracellular cAMP concentrations, and cAMP is vital for PKA activation^18,19^. These findings prompted us to investigate the role of the PKA/VLA-4/FAK signaling pathway in the therapeutic effects of WT161 on ALL.

This study investigated the therapeutic potential of WT161 in ALL, focusing on its impact on the VLA-4/FAK signaling pathway. Using integrated in vitro and in vivo approaches, we assessed the effects of WT161 on ALL cell proliferation, apoptosis, adhesion, and migration. In xenograft mouse models, we further evaluated its efficacy, both as a single agent and in combination with vincristine, a mainstay of ALL therapy. Our results demonstrate that WT161 suppresses ALL progression by inhibiting proliferation, inducing apoptosis, and impairing cell adhesion and migration. These effects were associated with reduced phosphorylation of VLA-4/FAK. Furthermore, WT161 enhanced the antitumor efficacy of vincristine and reduced tumor burden in vivo.

Given the high expression of HDAC6 in ALL and the selectivity of WT161, our study elucidates the mechanisms of WT161 action and provides a foundation for its clinical development as a novel treatment for ALL.

## Materials and methods

### Reagents and antibodies

WT161 was obtained from MedChemExpress (USA), vincristine from Abmole Bioscience Inc. (USA), fibronectin from Solarbio Life Sciences (China), and the CellTracker Green CMFDA (5-Chloromethylfluorescein Diacetate) from YEASEN (China). For flow cytometry, anti-CD49d-PE and anti-CD29-FITC were sourced from BioLegend (USA) and Thermo Fisher (USA), respectively.

The following antibodies were used for Western blot and other applications: HDAC6, β1 integrin, and α-tubulin (ABclonal, China); acetylated α-tubulin, α4 integrin, phospho-FAK, and phospho-EGFR (Affinity Biosciences, China); AP-conjugated α4 integrin (Sigma-Aldrich, China); FAK, EGFR, goat anti-rat IgG-HRP, and α-tubulin (Wanlei Biotechnology, China). Antibodies against PKA-Cα and phospho-PKA substrates were from Cell Signaling Technology (USA). The PKA inhibitor H-89 was purchased from Macklin Biotechnology Co., Ltd. (China).

### Cell culture and proliferation assay

Human ALL cell lines, including B-ALL lines (BALL-1 and NALM-6) and T-ALL lines (Jurkat and MOLT-4), were obtained from Qiyi Biotechnology Co., Ltd. (Shanghai, China). All cells were cultured in RPMI-1640 medium (Solarbio Life Sciences, China) supplemented with 10% fetal bovine serum, 2 mM L-glutamine, 100 U/mL penicillin, and 100 µg/mL streptomycin. Cells were maintained at 37 °C in a humidified incubator with 5% CO₂.

For the cell proliferation assay, cells in the logarithmic growth phase were harvested and seeded into 96-well plates. Following overnight adherence, the cells were treated with various concentrations of WT161 for 24, 48, and 72 h. At the indicated time points, 10 µL of CCK-8 reagent (Wanleibio, China) was added to each well, and the plates were incubated for an additional 2 h. The optical density (OD) at 450 nm was then measured using a microplate reader (BIOTEK, USA).

### Cell adhesion and migration assay

To evaluate cell adhesion, 96-well plates were coated with 10 µg/mL fibronectin and incubated overnight at 4 °C. Following treatment, cells were resuspended in PBS, stained with 2 µM CMFDA at 37 °C for 15 min, and seeded into fibronectin-coated wells at a density of 1 × 10⁵ cells per well. After 30 min of incubation at 37 °C, non-adherent cells were removed by washing three times with PBS. The fluorescence intensity of adherent cells was measured using a fluorescence microplate reader with excitation at 485 nm and emission at 520 nm. Each experiment was performed in triplicate.

Cell migration was assessed using Transwell inserts (Corning Costar; 6.5 mm diameter, 8 μm pore size). Cells were centrifuged at 150×g for 3 min and resuspended in serum-free RPMI-1640. The lower chamber of a 24-well plate was filled with 800 µL of RPMI-1640 containing 10% FBS as a chemoattractant, and 200 µL of cell suspension (5 × 10⁴ cells) was added to the upper chamber. After 24 h of incubation at 37 °C with 5% CO₂, migrated cells in the lower chamber were collected and counted.

### Flow cytometry analysis of cell cycle, apoptosis and surface markers

For cell cycle analysis, BALL-1, NALM-6, Jurkat, and MOLT-4 cells were treated with 5 µM WT161 for 72 h, harvested, and washed twice with PBS. Cells were fixed in 70% ethanol for at least 12 h at 4 °C. After fixation, cells were washed twice with PBS and stained in the dark with a solution containing 100 µL RNase and 500 µL propidium iodide (PI) for 30 min at 4 °C. Apoptosis was assessed using an Annexin V-FITC/PI apoptosis detection kit according to the manufacturer’s instructions. In parallel, the surface expression of CD49d and CD29 was evaluated using anti-CD49d-PE and anti-CD29-FITC antibodies, respectively. All samples were analyzed on a NovoCyte flow cytometer (ACEA Bio, USA), and data were processed using the corresponding analytical software.

### Quantitative PCR

Total RNA was extracted from cells using TRIpure reagent (RP1001, BioTeke, China), and RNA concentration was measured using a NanoDrop 2000 spectrophotometer (Thermo Fisher, USA). cDNA was synthesized from 1 µg total RNA by reverse transcription. qPCR was performed using a reaction mixture containing cDNA, forward and reverse primers, SYBR Green (Solarbio Life Sciences, China), Taq PCR Master Mix (Solarbio Life Sciences, China), and nuclease-free water. Amplification was carried out on an Exicycler 96 real-time PCR system (BIONEER, Korea). All reactions were run in technical triplicates, and relative gene expression was calculated using the 2–ΔΔCT method. Primer sequences used in this study are listed in Table 1.

**Table 1:** Primer sequences used for quantitative real-time PCR.

Gene	Sequences (5′–3′)	
CD29	GCACGATGTGATGATTTA	Forward
	CTTTGCTACGGTTGGTTA	Reverse
CD49d	TTTCGGTCTGATTCTGCT	Forward
	GAACTTCCTTGCCCTTAT	Reverse
HDAC6	GCGAAGAAGTAGGCAGAA	Forward
	GCAGTCCCACGATTAGG	Reverse
β-actin	GGCACCCAGCACAATGAA	Forward
	TAGAAGCATTTGCGGTGG	Reverse

### Western blot

Proteins were extracted from the cells, and their concentrations were determined using a standard curve. Total protein was separated via SDS-PAGE and transferred to polyvinylidene fluoride (PVDF) membranes. The membranes were blocked with 5% (w/v) skimmed milk powder in TBST for 1 h and then incubated with diluted primary antibodies overnight at 4 ℃. Following this, secondary antibodies were applied for 1 h at room temperature. Signals were detected using an ECL substrate, and the optical density of the target bands was analyzed using Gel-Pro Analyzer software.

### Caspase activity assay

Protein concentrations were measured using the Bradford Protein Concentration Determination Kit (Wanlei Biotechnology Co., Ltd., China), with a standard curve prepared to calculate the exact concentration. Caspase-3, -8, and − 9 activities were assessed using colorimetric assay kits (Beyotime Biotechnology, China) according to the manufacturer’s instructions. Briefly, cells were resuspended in cell lysis buffer, centrifuged, and the supernatants were collected. Samples were then incubated at 37 ℃ with reaction buffer and substrate for 1–2 h. Absorbance at 405 nm was measured using an ELX-800 enzyme marker (BIOTEK, USA) to calculate the amount of p-nitroaniline (pNA) produced through catalysis, with the calculation based on a pNA-specific standard curve.

### Immunofluorescence staining of ALL cells

β1-integrin activation in ALL cells was evaluated by immunofluorescence. Cells treated with or without 5 µM WT161 for 72 h were fixed with 4% formaldehyde for 10 min, permeabilized with 0.5% Triton X-100 in PBS for 15 min, and blocked with 1% BSA for 15 min. Samples were incubated overnight at 4 °C with a primary antibody against β1-integrin (clone 12G10; Abcam, ab30394, UK). After washing with PBS, cells were incubated for 1 h at room temperature with a FITC-conjugated goat anti-rabbit secondary antibody (Abcam, ab6717, UK). Nuclei were counterstained with DAPI (1:1000 dilution) for 5 min. Following final washes, cells were resuspended in antifade mounting medium, transferred to glass slides, and covered with coverslips. Images were acquired using an Olympus BX53 fluorescence microscope equipped with a DP73 camera system.

### Histological and Immunofluorescence analysis

For histological evaluation, paraffin-embedded tumor tissues were sectioned at 5 μm thickness. Hematoxylin and eosin (H&E) staining was performed to examine cellular morphology. For immunofluorescence, cryosections were fixed and incubated with an anti-acetyl-α-tubulin primary antibody, followed by a Cy3-conjugated goat anti-rabbit IgG secondary antibody (Invitrogen, USA) diluted 1:200 in PBS containing 1% BSA for 1 h at room temperature. Nuclei were counterstained with DAPI. Representative images from at least 10 independent fields were acquired using an Olympus DP73 fluorescence microscopy system (Japan).

### TUNEL assay

Apoptosis in tumor tissues was detected using a One Step TUNEL Apoptosis Assay Kit (Beyotime Biotechnology, China) following the manufacturer’s protocol.

### Animal studies

Twenty-four female NOD/SCID mice (6–8 weeks old) were obtained from Changzhou Cavens Experimental Animal Co., Ltd (Jiangsu, China). After one week of acclimation, the mice were randomly divided into two groups (*n* = 12 per group) and subcutaneously inoculated in the right axilla with 1 × 10⁷ BALL-1 or MOLT-4 cells, respectively. When tumor volumes reached approximately 200 mm³, each group was further divided into four subgroups (*n* = 3 per subgroup) receiving the following treatments: WT161 (80 mg/kg, i.p., daily), vincristine (1 mg/kg, i.p., weekly), their combination, or vehicle control. After 15 days of treatment, the mice were euthanized by CO₂ asphyxiation followed by cervical dislocation. Tumor tissues were collected, with portions fixed in 4% paraformaldehyde and the remainder snap-frozen and stored at − 70 °C for subsequent analysis. All animal experiments were approved by the Animal Ethics Committee of Guangdong Medical Experimental Animal Center (Guangdong, China) and conducted in accordance with institutional guidelines.

### Statistical analysis

Data are presented as the mean ± standard deviation (SD) from at least three independent experiments. Statistical comparisons between two groups were performed using Student’s t-test, while comparisons among multiple groups were analyzed by one-way ANOVA. A p-value of less than 0.05 was considered statistically significant. All statistical analyses were conducted using GraphPad Prism software (version 5.0; GraphPad Software, USA).

## Results

### WT161 suppresses ALL cells proliferation through α-tubulin acetylation

**Fig. 1:**
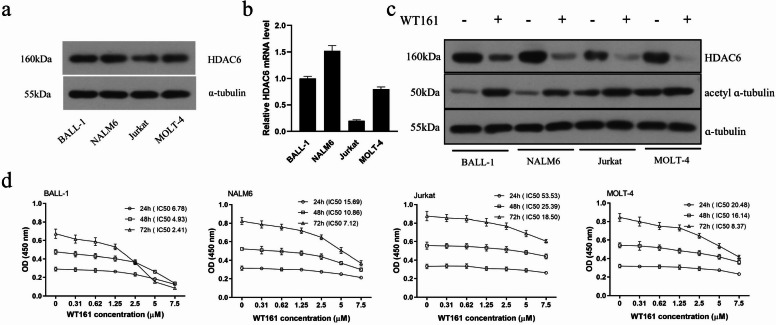
HDAC6 expression in ALL cell lines and the effect of WT161 on HDAC6, acetyl-α-tubulin, and cell proliferation. (**a**) HDAC6 protein expression in four ALL cell lines (BALL-1, NALM-6, Jurkat, and MOLT-4) as detected by Western blot. (**b**) Relative HDAC6 mRNA levels measured by quantitative PCR (qPCR), showing expression trends consistent with (**a**). (**c**) Western blot analysis of HDAC6 and acetyl-α-tubulin protein levels in cell lines treated with or without 5 µM WT161 for 72 h. (**d**) Concentration- and time-dependent inhibition of cell proliferation by WT161 across all four ALL cell lines. IC_50_ values were calculated to quantify compound sensitivity.

To evaluate the effect of the selective HDAC6 inhibitor WT161 on acute lymphoblastic leukemia (ALL), we first examined HDAC6 expression in four ALL cell lines (BALL-1, NALM-6, Jurkat, and MOLT-4). Western blot analysis revealed substantial HDAC6 protein expression in all cell lines, with Jurkat cells showing relatively lower levels (Fig. 1a). Consistently, quantitative PCR (qPCR) demonstrated a similar pattern at the transcriptional level, with Jurkat cells exhibiting the lowest HDAC6 mRNA expression (Fig. 1b). Treatment with 5 µM WT161 for 72 h significantly reduced HDAC6 expression while increasing acetylated α-tubulin levels (Fig. 1c), confirming effective HDAC6 inhibition by WT161 in ALL cells.

We next assessed the anti-proliferative effect of WT161. ALL cells treated with varying concentrations of WT161 for 24, 48, and 72 h showed a time- and dose-dependent suppression of proliferation (Fig. 1d). Notably, B-ALL cells were more sensitive to WT161 than T-ALL cells, as evidenced by their lower IC₅₀ values. Jurkat cells, which expressed the least HDAC6, consistently displayed the highest IC₅₀ values across all time points, indicating a correlation between HDAC6 expression levels and cellular sensitivity to WT161. These results demonstrate that WT161 effectively inhibits ALL cell proliferation, likely through modulation of HDAC6 activity and subsequent α-tubulin acetylation.

### WT161 triggers apoptosis and induces cell cycle arrest in ALL cells

**Fig. 2:**
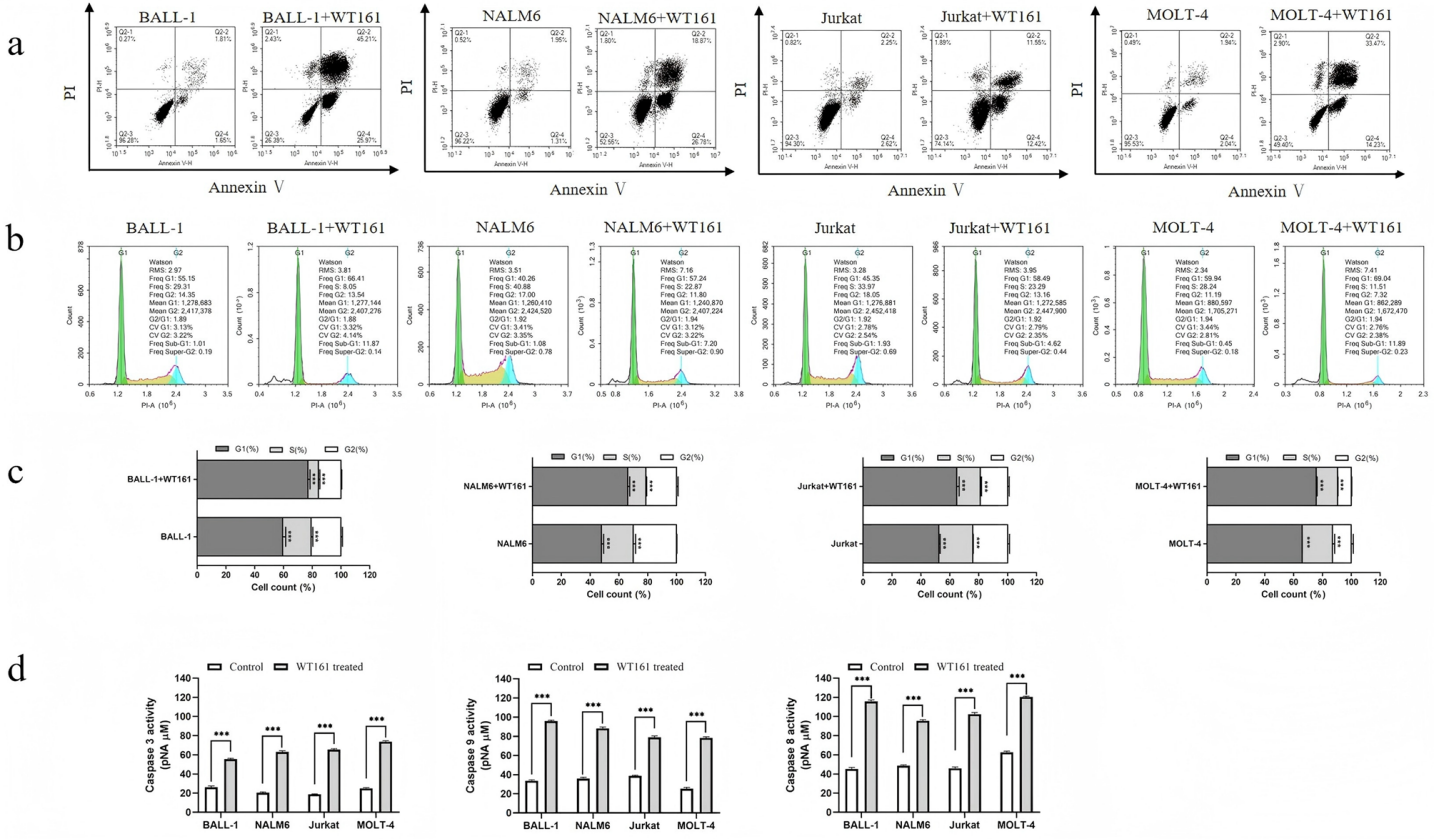
WT161 induces apoptosis and cell cycle arrest in ALL cells. (**a**) Apoptosis analysis by flow cytometry with Annexin V/PI staining in four ALL cell lines after treatment with 5 µM WT161 for 72 h. (**b**) Cell cycle distribution assessed by PI staining following WT161 treatment (5 µM, 72 h), showing G₁-phase accumulation and reduced S-phase population. (**c**) Quantitative analysis of the percentage of cells in G₁ and S phases. Data are presented as the mean ± SD (*n* = 3). ***P < 0.001 vs. control. (**d**) Caspase-3, -8, and − 9 activity in ALL cells treated with WT161 (5 µM, 72 h). ***P < 0.001.

To further explore the mechanism underlying the anti-proliferative effect of WT161, we examined its role in apoptosis and cell cycle regulation. Flow cytometric analysis with Annexin V/PI staining demonstrated that treatment with 5 µM WT161 for 72 h significantly increased apoptosis across all ALL cell lines (Fig. 2a). Cell cycle analysis revealed a marked accumulation of cells in the G₁ phase, accompanied by a reduction in the proportion of cells in S phase (Fig. 2b and c). Notably, a significant increase in the sub-G₁ population was observed in all WT161-treated cell lines, consistent with enhanced apoptotic activity (Fig. 2b). Furthermore, caspase activity assays showed elevated levels of Caspase-3, -8, and − 9, indicating activation of both intrinsic and extrinsic apoptotic pathways (Fig. 2d). Collectively, these results demonstrate that WT161 exerts anti-leukemic effects by inducing cell cycle arrest at the G₁ phase and promoting apoptosis in ALL cells.

### WT161 suppresses ALL cell adhesion, migration, and VLA-4 expression

**Fig. 3:**
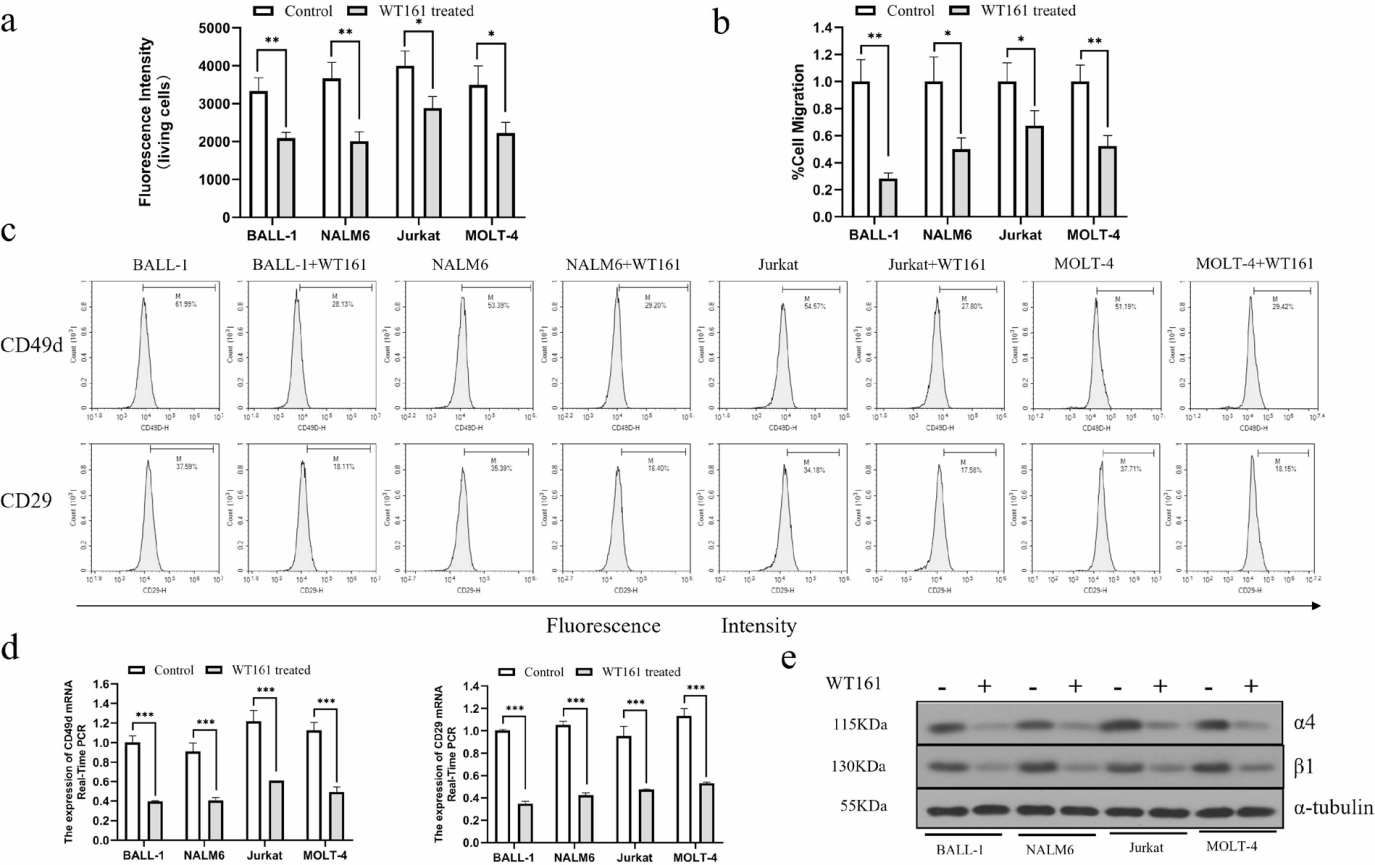
WT161 impairs adhesion, migration, and VLA-4 expression in ALL cells. (**a**) Adhesion assay results showing reduced adhesion capacity of ALL cells after treatment with 5 µM WT161 for 72 h, as measured by fluorescence intensity. (**b**) Transwell migration assay demonstrating decreased migration ability of ALL cells treated with 5 µM WT161 for 72 h, as indicated by fewer cells migrating to the lower chamber. (**c**) Flow cytometry analysis showing downregulated surface expression of VLA-4 subunit (CD49d and CD29) in ALL cells treated with WT161 (5 µM, 72 h). (**d**) qRT-PCR data showing the decreased mRNA level of VLA-4 subunits (CD49d/CD29) in ALL cells in response to 5 µM WT161 (72 h treatment). (**e**) Protein expression of VLA-4 subunits assessed by Western blot in ALL cells following WT161 treatment (5 µM, 72 h). Data are presented as mean ± SD (*n* = 3). *P < 0.05, **P < 0.01, ***P < 0.001 vs. control.

We next evaluated the effect of WT161 on cellular adhesion and migration. Adhesion assays showed that treatment with 5 µM WT161 for 72 h significantly reduced adhesion in all tested cell lines, with B-ALL cells (BALL-1 and NALM-6, *P* < 0.01) exhibiting greater sensitivity than T-ALL cells (Jurkat and MOLT-4, *P* < 0.05) (Fig. 3a). Similarly, Transwell migration assays revealed a marked decrease in migrated cells upon WT161 treatment, further confirming its inhibitory effect on cell motility (Fig. 3b). Among the four lines, Jurkat cells showed the highest baseline migration rate.

Given the pivotal role of VLA-4 in lymphocyte adhesion and migration, we examined whether WT161 modulates its expression. Flow cytometry analysis demonstrated that WT161 significantly reduced surface expression of the VLA-4 subunits CD49d and CD29 (Fig. 3c and Figure S1). Consistent with this, both mRNA and protein levels of CD49d and CD29 were notably downregulated following WT161 treatment (Fig. 3d and e). These results collectively indicate that WT161 impairs ALL cell adhesion and migration, at least in part, through suppression of VLA-4 expression.

### WT161 suppresses PKA activity and modulates VLA-4 signaling in ALL cells

**Fig. 4:**
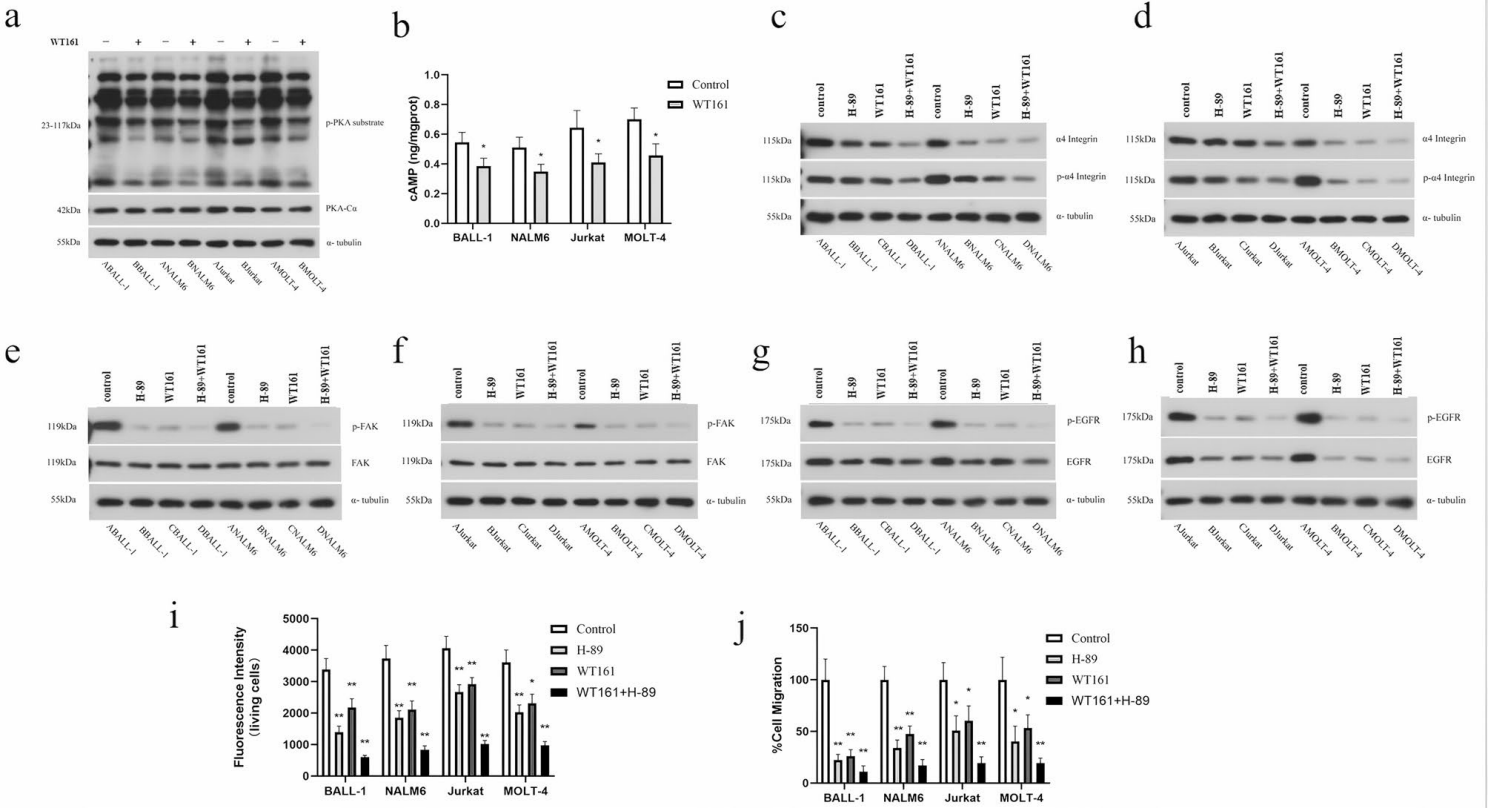
WT161 suppresses PKA signaling and impairs ALL cell adhesion and migration. (**a**) Western blot analysis of phospho-PKA substrates and PKA-Cα in ALL cells treated with 5 µM WT161 for 72 h. (**b**) Intracellular cAMP levelsmeasured by ELISA in WT161-treated cells (5 µM, 72 h). (**c**,**d**) Expression and phosphorylation of α4-integrin at Ser988 in (**c**) B-ALL and (**d**) T-ALL cells treated with WT161 (5 µM), H-89 (PKA inhibitor), or their combination for 72 h. (**e**,**f**) FAK and phospho-FAK (Tyr397) levels in (**e**) B-ALL and (**f**) T-ALL cells under the same treatment conditions. (**g**,**h**) EGFR and phospho-EGFR (Tyr1068) expression in (**g**) B-ALL and (**h**) T-ALL cells. (**i**) Quantification of adherent cells by fluorescence intensity. (**j**) Ratio of migrated cells (measured by Transwell assay) showing decreased migration. Data are presented as mean ± SD (*n* = 3). *P < 0.05, **P < 0.01, ***P < 0.001 vs. control.

Based on previous reports that HDAC6 inhibition reduces intracellular cAMP levels^18^, we first evaluated the effect of WT161 on PKA activity. Phospho-PKA substrate motif analysis showed that WT161 significantly decreased PKA substrate phosphorylation (Fig. 4a). Consistent with this, ELISA measurements confirmed a marked reduction in intracellular cAMP levels following WT161 treatment (Fig. 4b), indicating that WT161 suppresses PKA activation through cAMP downregulation.

To determine whether WT161 affects VLA-4 via PKA signaling, we treated BALL-1, NALM-6, Jurkat, and MOLT-4 cells with the PKA inhibitor H-89 alone, WT161 alone, or their combination. Both H-89 and WT161 individually reduced phosphorylation at Ser988 and expression of integrin α4 (CD49d) across all cell lines, with the most pronounced reduction observed in the combination treatment group (Fig. 4c and d). Since Ser988 phosphorylation by PKA activates VLA-4, these results suggest synergistic inhibition of VLA-4 activation.

We further examined the downstream FAK signaling pathway. While neither H-89 nor WT161 affected total FAK protein levels, both treatments significantly reduced FAK phosphorylation at Tyr397, with the strongest suppression again seen in the combination group (Fig. 4e and f). Similarly, EGFR protein expression and phosphorylation at Tyr1068 were downregulated in a comparable pattern to integrin α4 (Fig. 4g and h).

To functionally validate these findings, we assessed cell adhesion and migration under the same treatment conditions. H-89, WT161, and their combination all significantly reduced adherent cell numbers (Fig. 4i) and migration rates (Fig. 4j) compared to controls. In B-ALL cells, individual treatments strongly inhibited migration (*P* < 0.01), whereas in T-ALL cells, significant inhibition required combination treatment (*P* < 0.05).

**Fig. 5:**
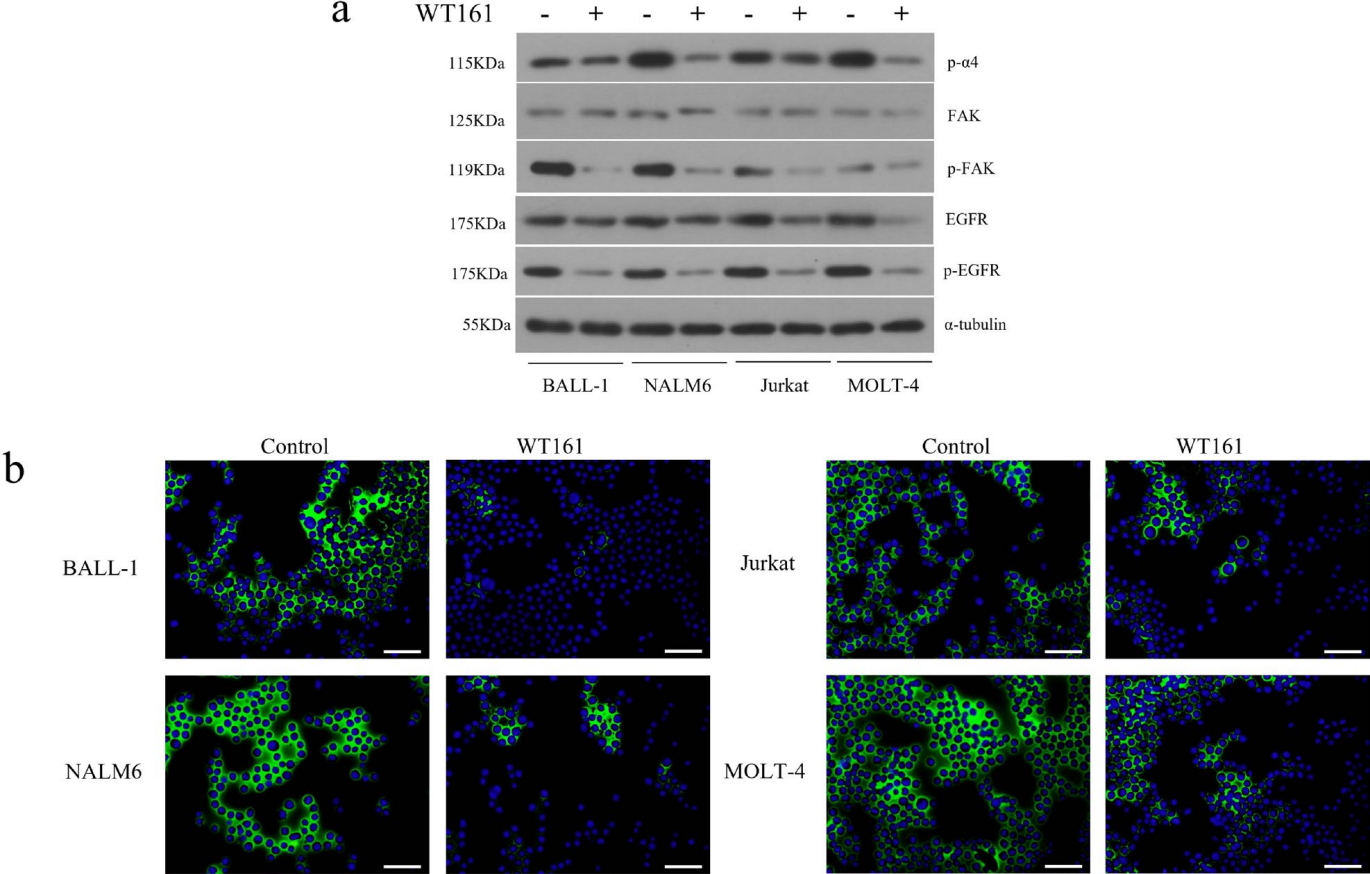
WT161 suppresses the VLA-4/FAK signaling pathway in ALL cells. (**a**) Western blot analysis of phosphorylated α4-integrin (Ser988), FAK (Tyr397), and EGFR (Tyr1068) in ALL cells treated with 5 µM WT161 for 72 h. (**b**) Immunofluorescence staining of active β1 integrin (green) and nuclei (blue, DAPI) in ALL cells following WT161 treatment (5 µM, 72 h), showing reduced membrane localization of active β1 integrin.

To further confirm WT161’s effect on VLA-4/FAK pathway activity, we analyzed key phosphorylation events in all four cell lines. WT161 significantly reduced phosphorylation of α4-integrin (Ser988), FAK (Tyr397), and EGFR (Tyr1068), as well as total EGFR protein expression (Fig. 5a). Immunofluorescence staining of active β1-integrin revealed that WT161 decreased β1-integrin membrane localization in all cell lines, with T-ALL cells maintaining higher residual activity than B-ALL cells (Fig. 5b).

Collectively, these results demonstrate that WT161 inhibits PKA activity by reducing intracellular cAMP levels, leading to suppression of the VLA-4/FAK signaling pathway and consequent impairment of ALL cell adhesion and migration capabilities.

### WT161 suppresses tumor growth and enhances vincristine efficacy in a murine ALL model

To evaluate the anti-tumor activity of WT161 in vivo, we established a xenograft mouse model and examined its effects on tumor growth and apoptosis. Given that HDAC6 regulates microtubule dynamics through deacetylation and that vincristine, a cornerstone ALL chemotherapeutic, targets microtubules, we hypothesized that WT161 might synergize with vincristine by modulating microtubule stability.

**Fig. 6:**
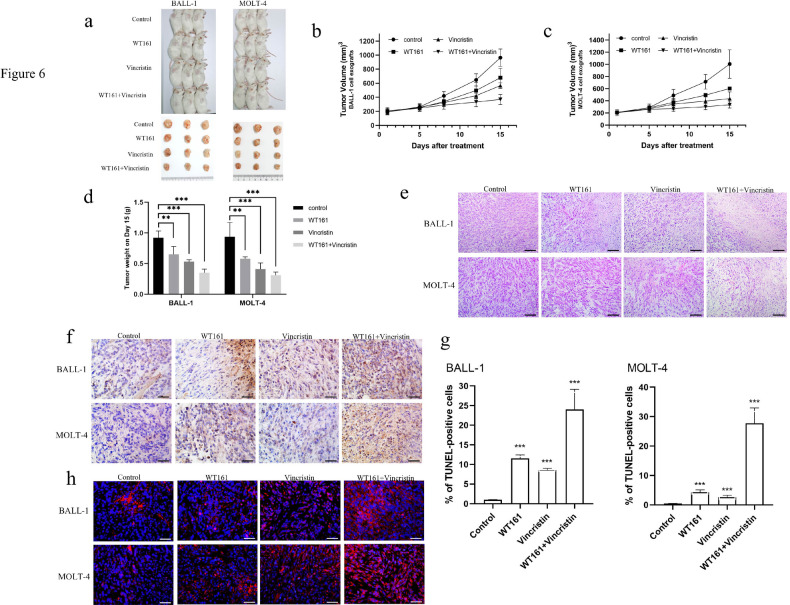
WT161 suppresses tumor growth and enhances vincristine efficacy in ALL xenograft models. (**a**–**c**) Tumor volume changes in mice treated with WT161 (80 mg/kg, daily), vincristine (1 mg/kg, weekly), or their combination for 15 days. (**d**) Tumor weight at endpoint showing reduced tumor burden, particularly in the combination group. (**e**) H&E staining of tumor sections revealing increased necrosis, nuclear condensation, and decreased cellular density in treated groups. (**f**) TUNEL staining demonstrating enhanced apoptosis in tumor tissues after treatment. (**g**) Quantitative analysis of TUNEL-positive cells confirming the pro-apoptotic effect of WT161 in vivo. (**h**) Immunofluorescence staining showing increased acetylated α-tubulin (red) in tumor tissues following WT161 treatment. Data are presented as mean ± SD (*n* = 3). **P < 0.01, ***P < 0.001 vs. control.

After 15 days of treatment, significant reductions in tumor volume (Fig. 6a and c) and weight (Fig. 6d) were observed in mice receiving WT161 alone, vincristine alone, or their combination. While both monotherapies inhibited tumor growth, the combination therapy yielded the most pronounced anti-tumor effect. Histological examination by H&E staining revealed substantial tumor cell necrosis and reduced viable tumor area in all treatment groups compared to controls (Fig. 6e). TUNEL assays further confirmed that the WT161-vincristine combination induced tumor cell apoptosis more effectively than either agent alone (Fig. 6f and g). Immunofluorescence analysis of tumor sections showed markedly elevated α-tubulin acetylation following combination treatment (Fig. 6h), indicating enhanced microtubule stabilization.

These results demonstrate that WT161 not only inhibits ALL proliferation and promotes apoptosis in vivo via α-tubulin hyperacetylation but also synergizes with vincristine, supporting its potential as a combination therapy for ALL.

## Discussion

ALL remains a considerable therapeutic challenge, especially in adults. The pronounced genetic heterogeneity of ALL underscores the critical need to elucidate its molecular mechanisms to develop more effective treatments.

Epigenetic alterations in cancer development critically regulate gene expression through post-translational modifications, such as acetylation, methylation, and phosphorylation^20^. Among these, histone acetylation and histone deacetylases (HDACs) have been extensively studied. HDACs modulate gene expression by removing acetyl groups from lysine residues of on histones and other proteins. Several HDACi drugs—including vorinostat, romidepsin, panobinostat and belinostat—have been developed to target neoplastic cells. However, clinical trials of these agents have demonstrated unsatisfactory safety and efficacy in cancer treatment^21^. HDAC6 is distinct from other HDACs due to its unique functions and substrates. HDAC6 is localized mainly in the cytoplasm, with non-histone proteins substrates including α-tubulin, cortactin, and heat shock protein 90 (HSP 90). By deacetylating α-tubulin, HDAC6 plays a critical role in the growth, progression, and migration of tumor cells through regulating cytoskeletal dynamics^22,23^. WT161 is a novel HDAC6 inhibitor that exhibits synergistic antitumor effects with cisplatin^12^, bortezomib^13^, 5-Fluorouracil (5-FU)^24^, and the small molecular inhibitor OTX015^25^.

Previous studies have reported HDAC6 overexpression in lymphoid cells^26^. Accordingly, we began by determining HDAC6 expression in the BALL-1, NALM-6, Jurkat, and MOLT-4 cell lines. High HDAC6 expression at both the protein and mRNA levels identified it as a promising therapeutic target. Notably, HDAC6 expression was significantly lower in Jurkat cells than in the other three lines. We subsequently investigated the effects of the selective HDAC6 inhibitor WT161 on these ALL cells, focusing on its impact on proliferation, apoptosis, adhesion, migration, and the VLA-4/FAK signaling pathway. Our results demonstrated that WT161 significantly inhibited the proliferation of ALL cells in a dose- and time-dependent manner. WT161 induces apoptosis in ALL cells, as confirmed by increased caspase activity and G1- phase cell cycle arrest. The inhibitory effects of WT161 on cell adhesion and migration were more potent in B-ALL cells than in T-ALL cells. This disparity was associated with higher HDAC6 expression levels in B-ALL cells, and the correspondingly higher IC_50_ values in T-ALL cells further indicated a differential sensitivity to WT161, warranting further investigation. Mechanistically, WT161 treatment elevated acetylated α-tubulin levels, thereby enhancing microtubule stability. This stabilization can cause cells to arrest during mitosis^27,28^, triggering cellular stress responses and activating apoptotic signaling pathways^29,30^. Enhanced microtubule stability likely promotes the expression and activation of pro-apoptotic factors, including cytochrome c release and caspase activation^31^. These findings highlight the pro-apoptotic potential of WT161, suggesting that it could enhance the therapeutic efficacy of existing treatments. The involvement of caspases in mediating the apoptotic response further emphasizes the need to explore combination therapies that synergize with WT161 to promote cell death in ALL. The adhesion and migration of ALL cells significantly contribute to their malignancy. These cells adhere to bone marrow stromal cells and other immune cells, receiving survival signals that help them evade apoptosis and enhance their viability^32,33^. Given that VLA-4 is crucial for the adhesion of leukemic cells to the bone marrow microenvironment, reducing its expression and activity could disrupt the protective niche that supports their survival and proliferation. Increased migration allows ALL cells to disseminate from the primary site to other tissues and organs, exacerbating disease severity and leading to challenging complications^34^. Integrins and focal adhesion kinase (FAK) signaling are essential for the metastasis of ALL cells, influencing cytoskeletal rearrangements that are critical for their motility. Therefore, the inhibition of cell adhesion and migration by WT161 presents a promising therapeutic strategy for treating ALL.

Notably, suppression of the PKA signaling pathway emerged as a critical mechanism mediating WT161’s effects. WT161 reduced intracellular cAMP levels, thereby inhibiting PKA activity. As a pivotal kinase, PKA drives ALL progression through multiple mechanisms^35,36^. PKA phosphorylates c-Myc, enhancing its stability and promoting the transcription of genes associated with cell proliferation, thereby facilitating the growth of ALL cells^37^. Furthermore, PKA upregulates Bcl-2 expression^38,39^, enhancing its anti-apoptotic activity, and enabling ALL cells to evade apoptosis more effectively, thereby promoting cell survival.

 In vivo studies corroborated the clinical relevance of our findings, demonstrating that WT161 not only inhibits tumor growth but also synergized with vincristine, a cornerstone of ALL treatment. This synergy suggest that WT161 may counteract vincristine resistance mechanisms, potentially by altering microtubule dynamics and enhancing apoptosis beyond the efficacy of either single agent. Although selective HDAC6 inhibitors, such as TubA, ACY-1215 (ricolinostat), Tubacin and ACY-738, have demonstrated preclinical efficacy, their clinical effectiveness remains unproven due to a lack of large-scale randomized controlled trials^40^. The efficacy of these inhibitors in real-world clinical settings remains uncertain.

Our study elucidates the multi-faceted anti-leukemic effects of the selective HDAC6 inhibitor WT161, encompassing the suppression of proliferation, induction of apoptosis, and disruption of VLA-4/FAK signaling. The high expression of HDAC6 in lymphocytes underpins WT161’s selectivity. Its significant anti-tumor effects, demonstrated in both in vitro and in vivo models, along with a tolerable safety profile in mouse xenograft studies, provides a strong rationale for advancing WT161 into clinical trials, particularly in combination with vincristine. Future research should focus on exploring the clinical applicability of this combination therapy and investigate the underlying mechanisms to optimize treatment strategies for ALL patients. A key direction will be to identify the specific downstream effectors of PKA, such as Bcl-2 family proteins or c-Myc, which are implicated in its critical mediating role.

## Supplementary Information

Below is the link to the electronic supplementary material.

Supplementary Material 1

Supplementary Material 2

Supplementary Material 3

## Data Availability

All data generated or analysed during this study are included in this published article.

## Abbreviations

ALL: Acute lymphoblastic leukemia
HDACs: Histone deacetylases
FAK: Focal adhesion kinase
OD: Optical density
