# Supplementary material for: Selective HDAC6 inhibitor WT161 modulates the VLA-4/FAK pathway by inhibiting PKA activity in acute lymphoblastic leukemia
Source: Sci Rep. 2025 Nov 17;15:40178. doi: 10.1038/s41598-025-23887-y (PMC12624111; doi:10.1038/s41598-025-23887-y)

Figure 1a

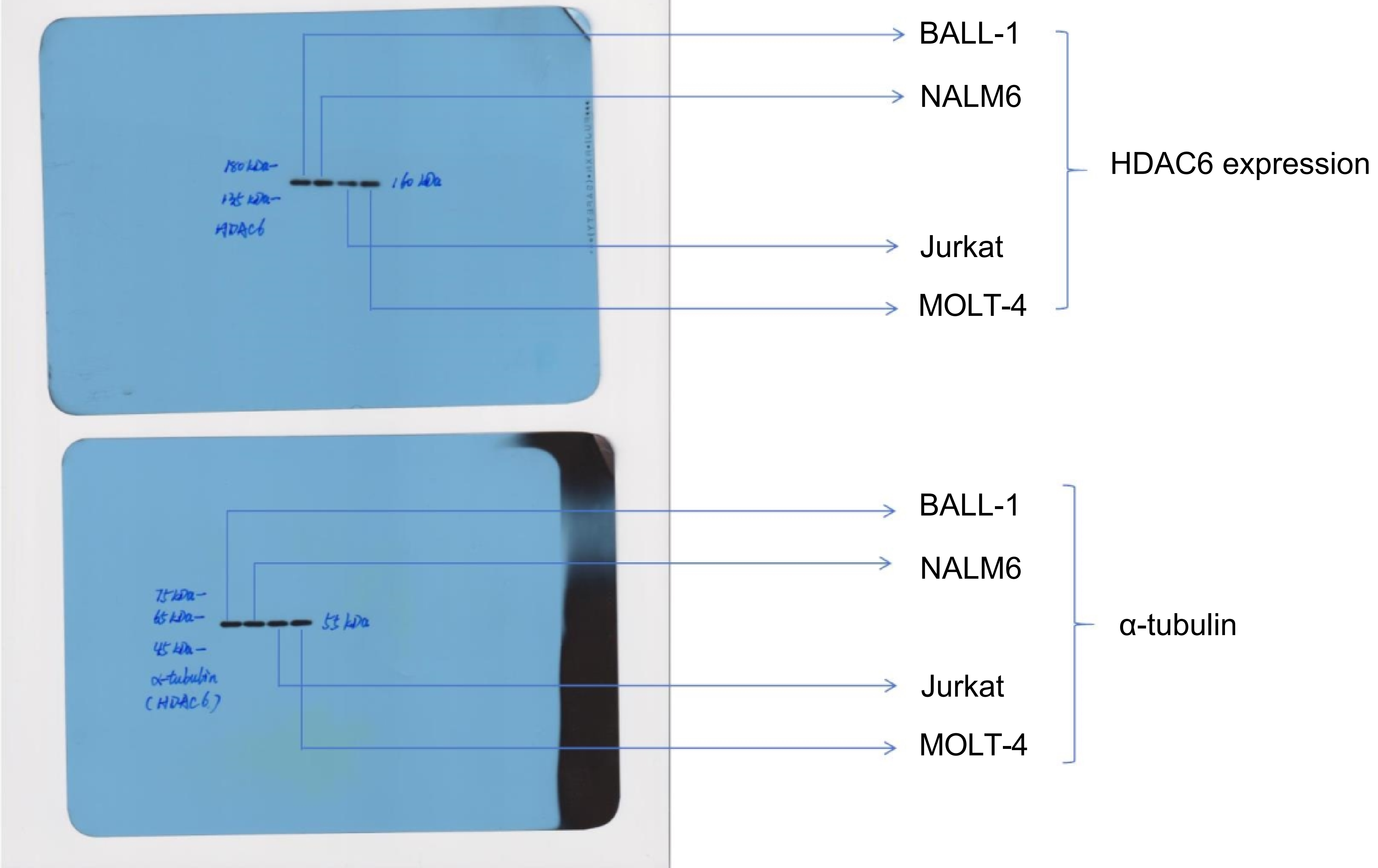
BALL-1
NALM6
Jurkat
MOLT-4
HDAC6 expression
BALL-1
NALM6
Jurkat
MOLT-4
α-tubulin

Figure 1c

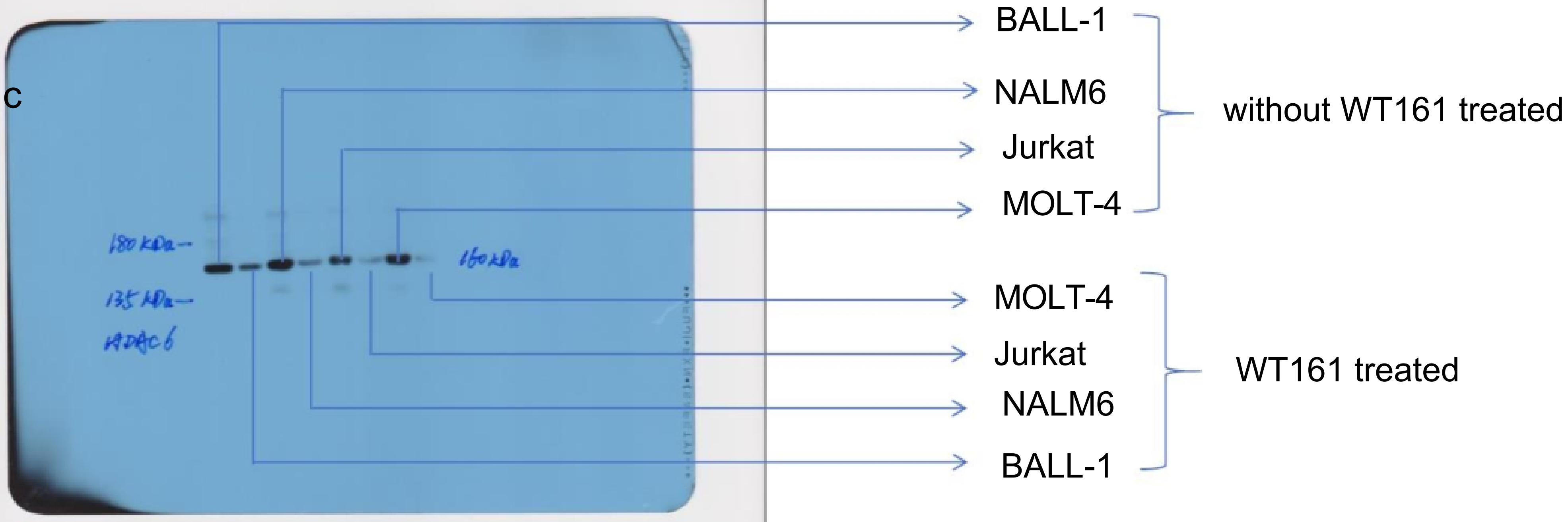

Figure 1c

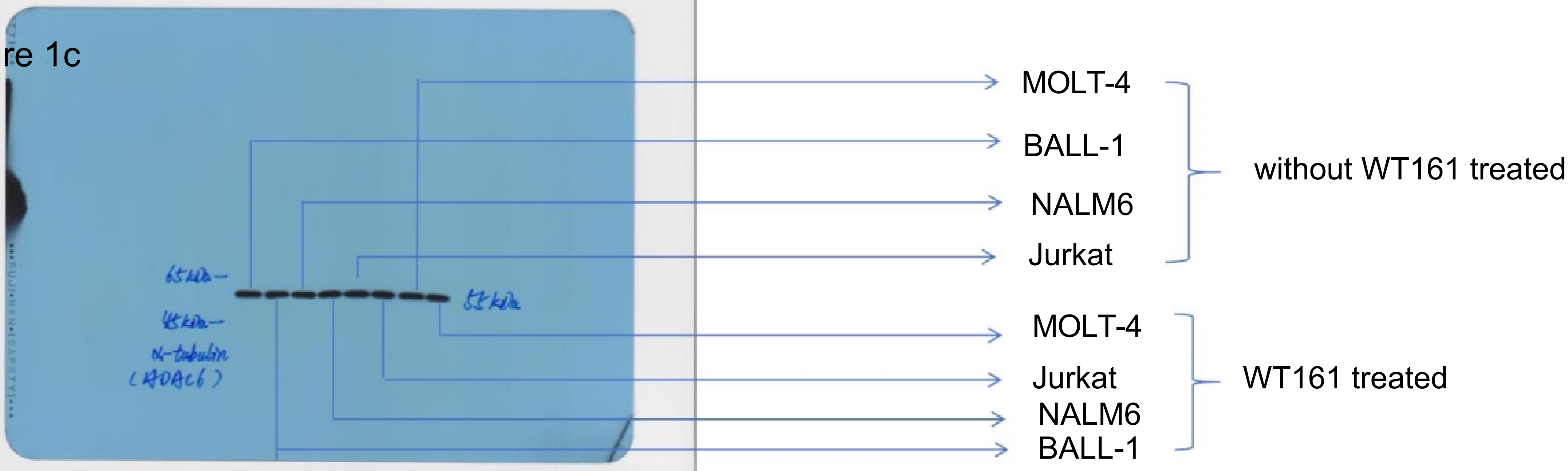

Figure 1c

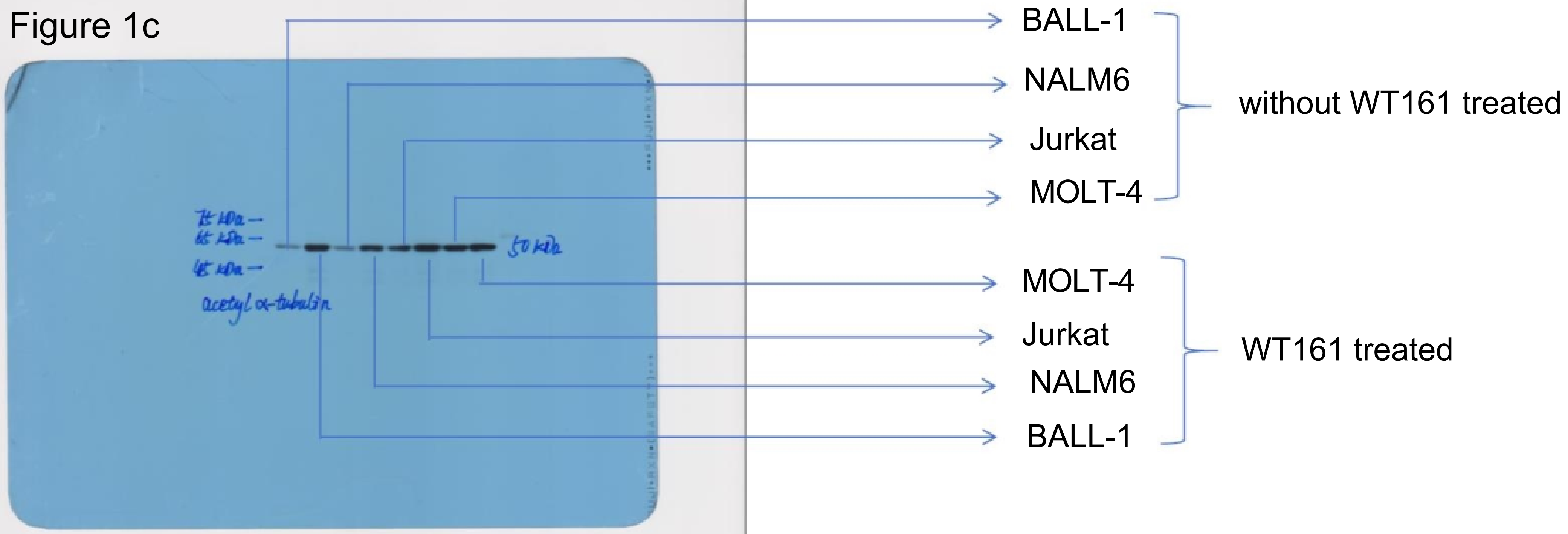

Figure 1c

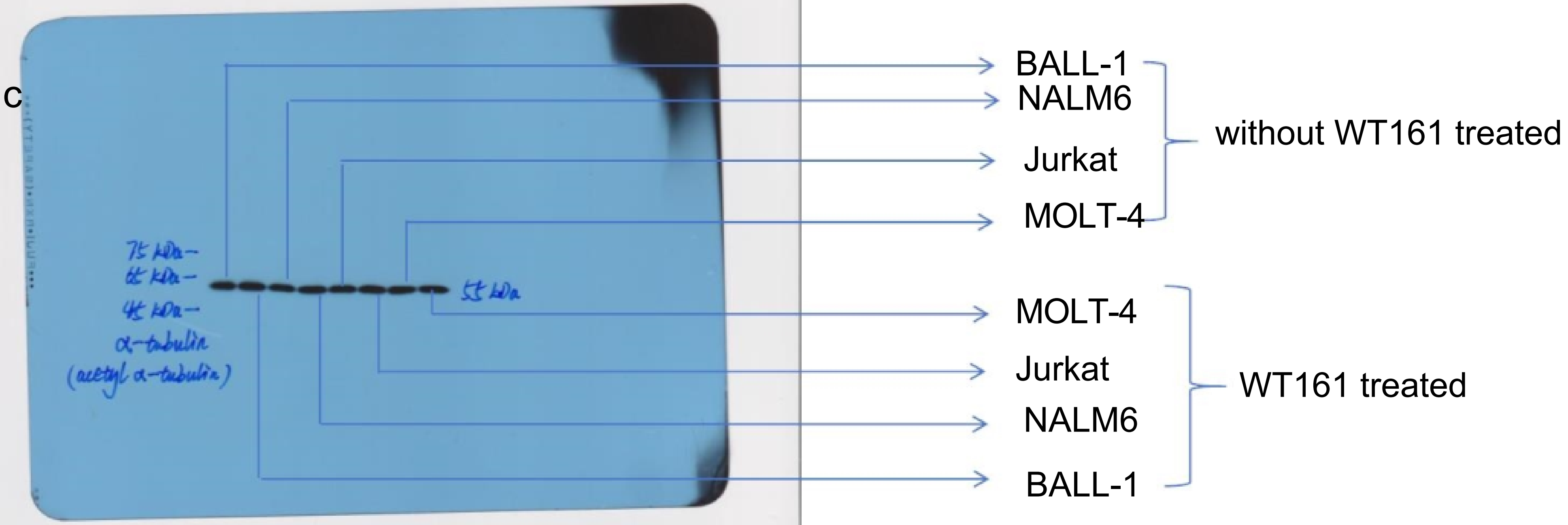

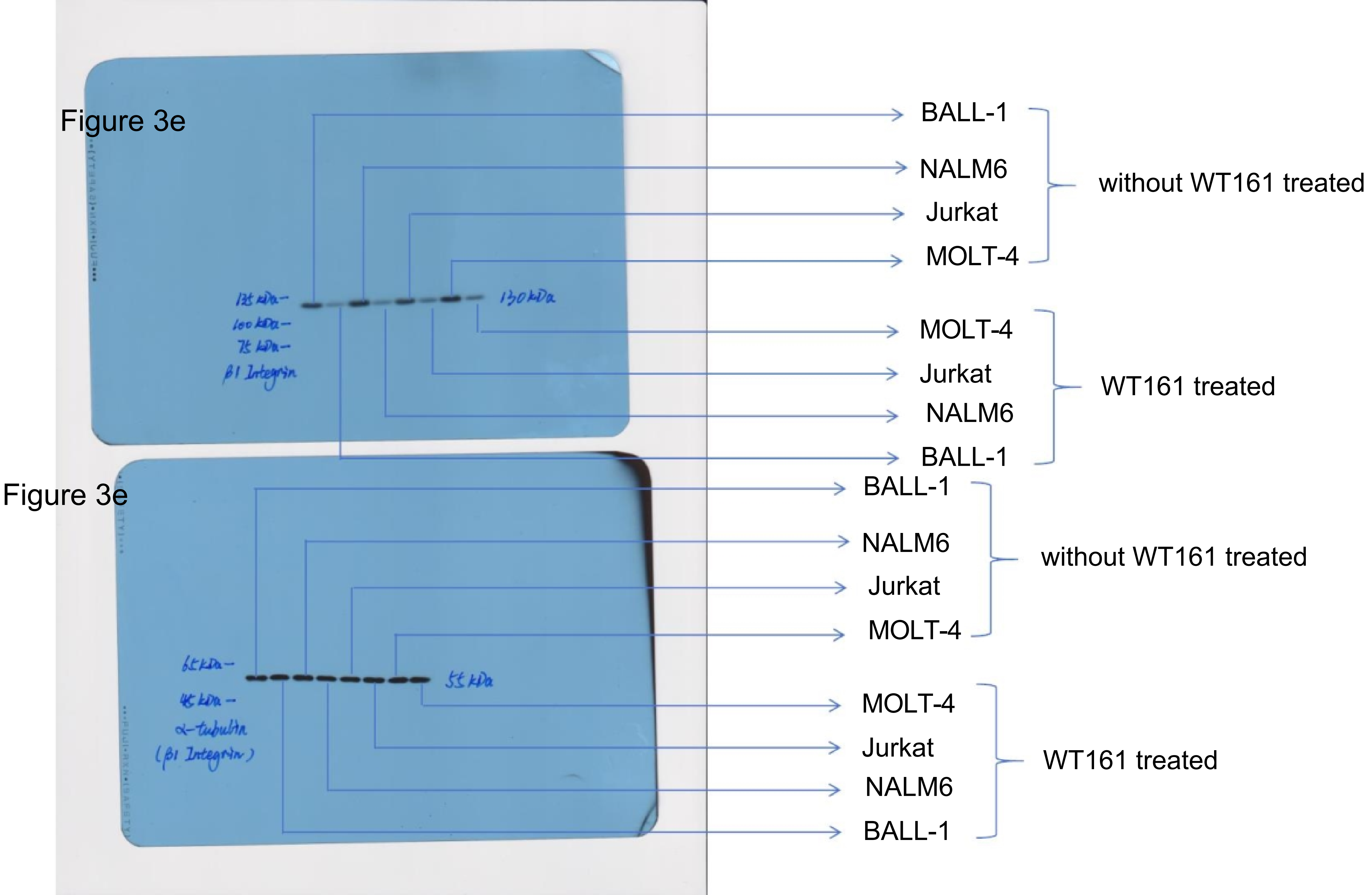
Figure 3e
135 kDa-
100 kDa-
75 kDa-
β1 Integrin
130 kDa
BALL-1
NALM6
Jurkat
MOLT-4
without WT161 treated
MOLT-4
Jurkat
NALM6
BALL-1
WT161 treated
Figure 3e
65 kDa-
45 kDa-
α-tubulin
(β1 Integrin)
55 kDa
BALL-1
NALM6
Jurkat
MOLT-4
without WT161 treated
MOLT-4
Jurkat
NALM6
BALL-1
WT161 treated

Figure 4a

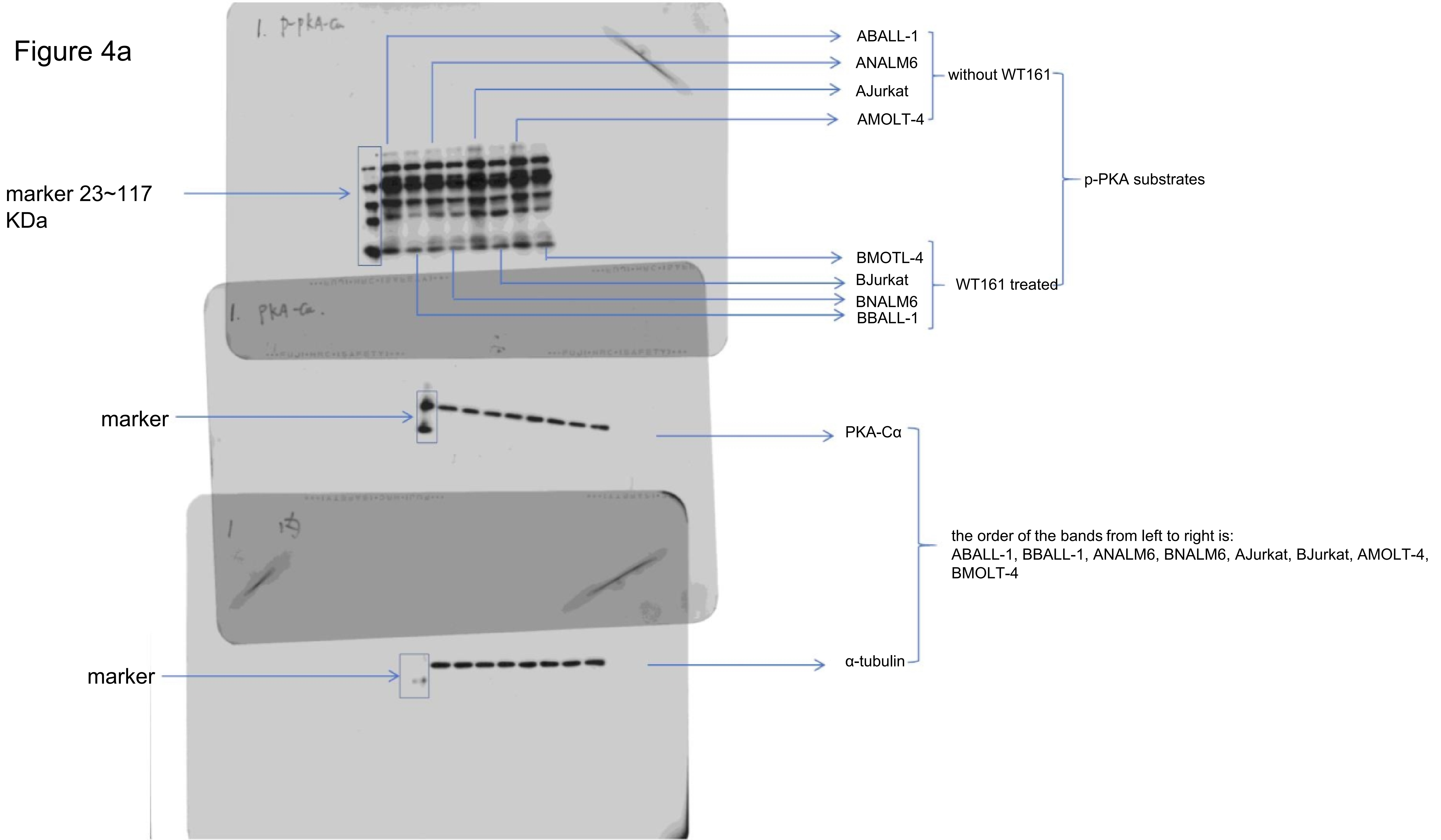
marker 23~117 KDa
ABALL-1
ANALM6
AJurkat
AMOLT-4
without WT161
p-PKA substrates
BMOTL-4
BJurkat
BNALM6
BBALL-1
WT161 treated
marker
PKA-Cα
the order of the bands from left to right is:
ABALL-1, BBALL-1, ANALM6, BNALM6, AJurkat, BJurkat, AMOLT-4, BMOLT-4
marker
α-tubulin

Figure 4c

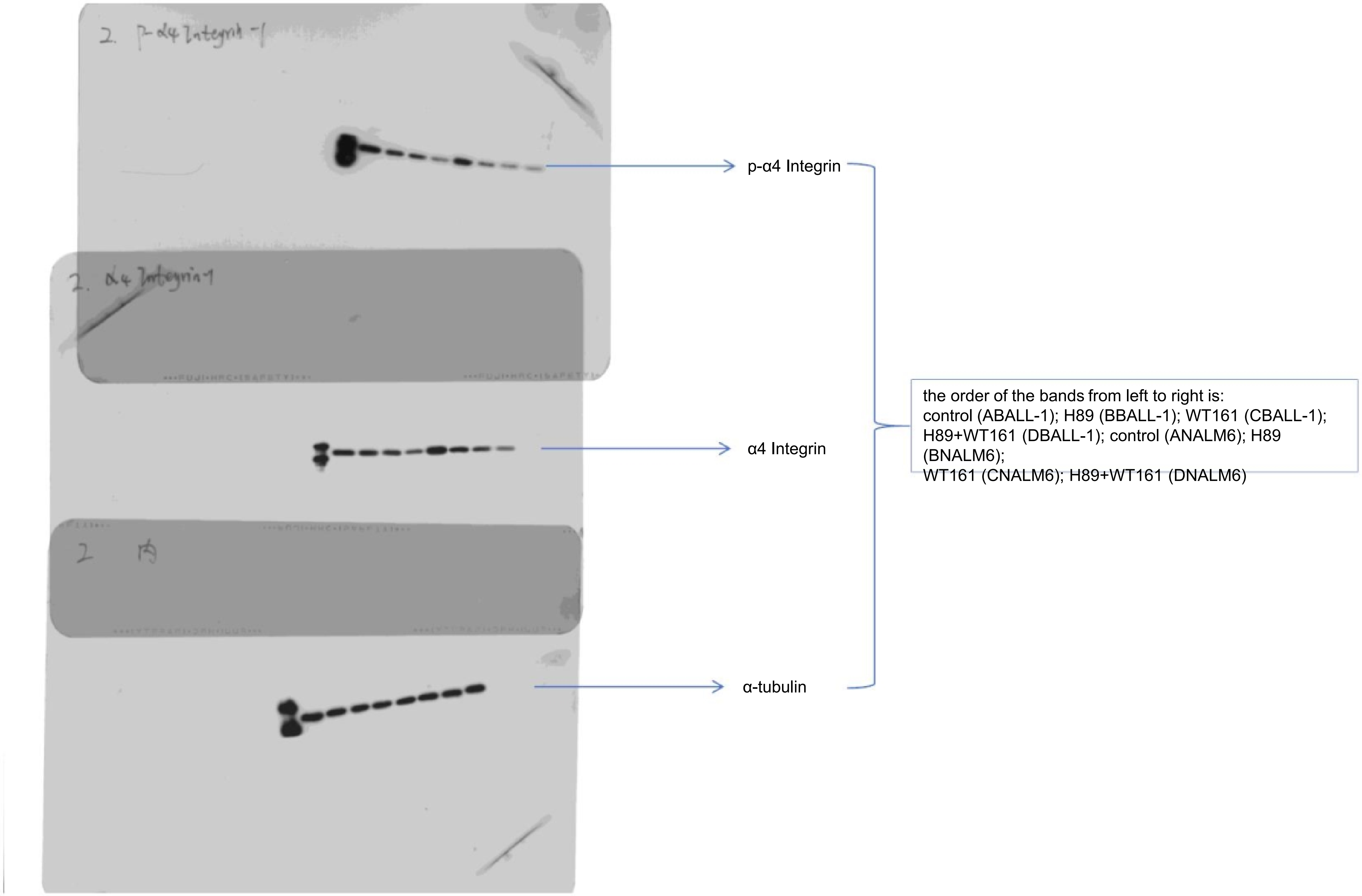
p-α4 Integrin
α4 Integrin
α-tubulin
the order of the bands from left to right is:
control (ABALL-1); H89 (BBALL-1); WT161 (CBALL-1); H89+WT161 (DBALL-1); control (ANALM6); H89 (BNALM6);
WT161 (CNALM6); H89+WT161 (DNALM6)

Figure 4d

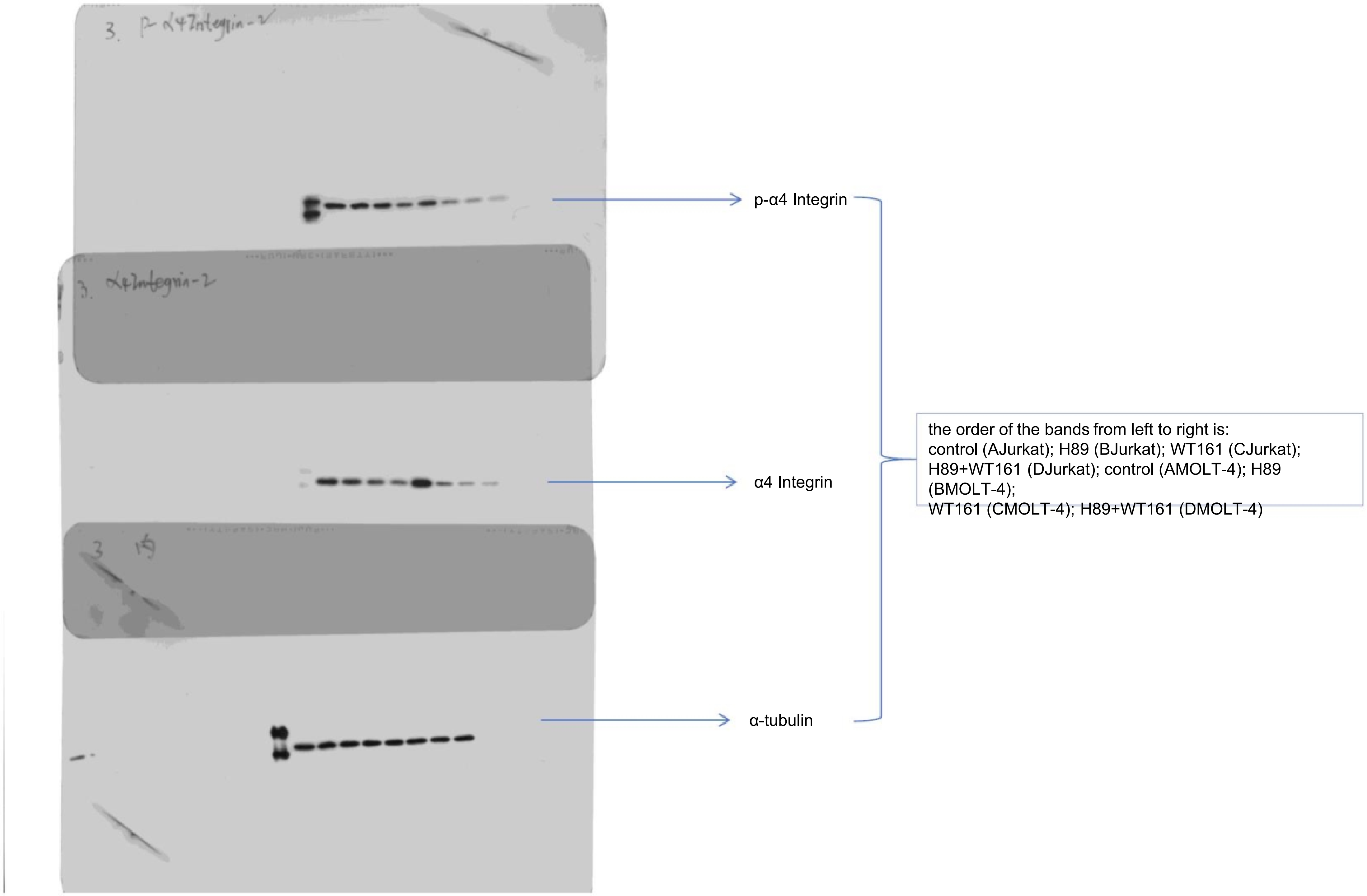
p-α4 Integrin
α4 Integrin
α-tubulin
the order of the bands from left to right is:
control (AJurkat); H89 (BJurkat); WT161 (CJurkat); H89+WT161 (DJurkat); control (AMOLT-4); H89 (BMOLT-4);
WT161 (CMOLT-4); H89+WT161 (DMOLT-4)

Figure 4e

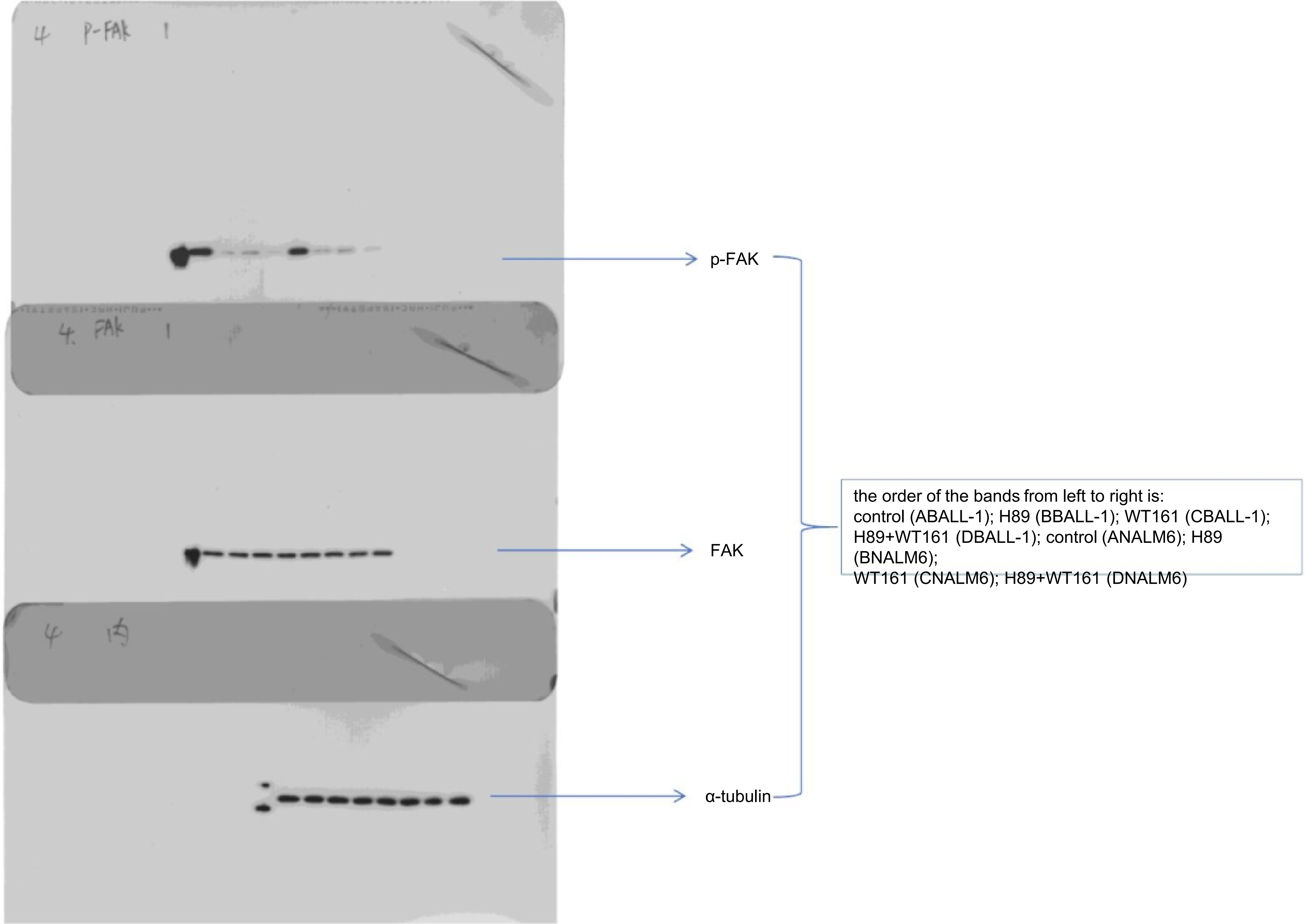
4 p-FAK 1
4. FAK 1
4 内
p-FAK
FAK
α-tubulin
the order of the bands from left to right is:
control (ABALL-1); H89 (BBALL-1); WT161 (CBALL-1);
H89+WT161 (DBALL-1); control (ANALM6); H89
(BNALM6);
WT161 (CNALM6); H89+WT161 (DNALM6)

Figure 4f

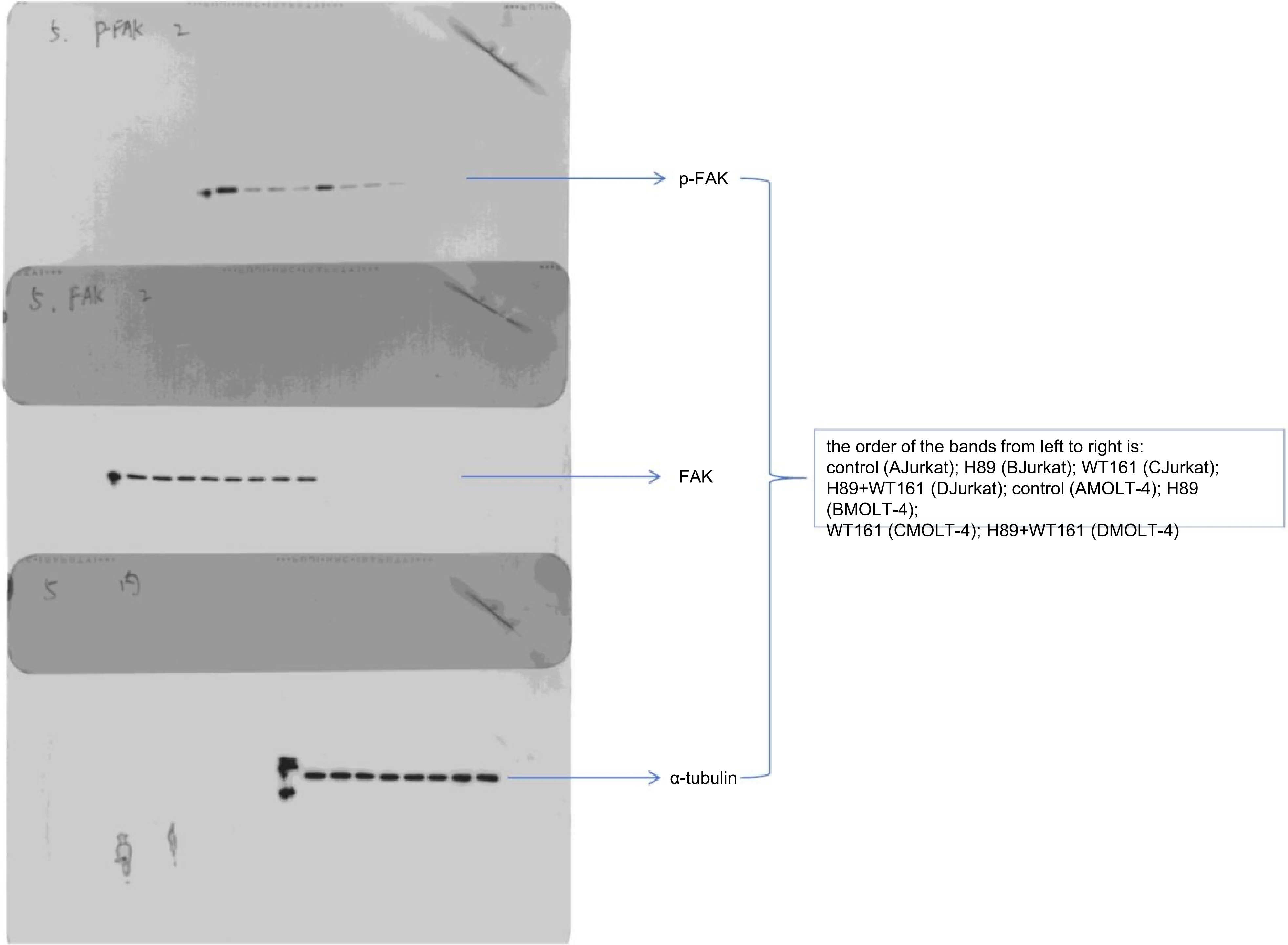
p-FAK
FAK
α-tubulin
the order of the bands from left to right is:
control (AJurkat); H89 (BJurkat); WT161 (CJurkat); H89+WT161 (DJurkat); control (AMOLT-4); H89 (BMOLT-4);
WT161 (CMOLT-4); H89+WT161 (DMOLT-4)

Figure 4g

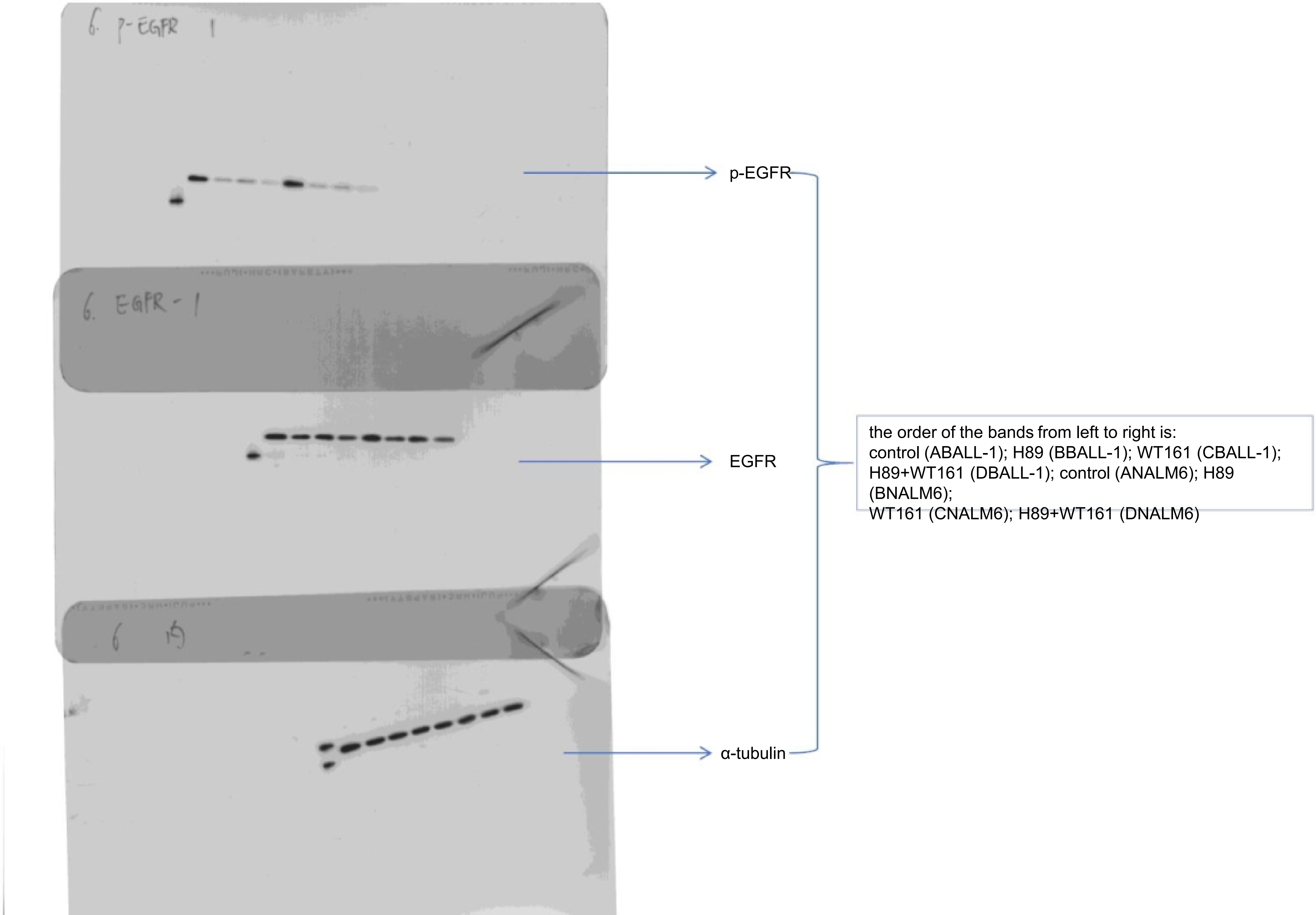
6. p-EGFR 1
6. EGFR-1
6 15
p-EGFR
EGFR
α-tubulin
the order of the bands from left to right is:
control (ABALL-1); H89 (BBALL-1); WT161 (CBALL-1);
H89+WT161 (DBALL-1); control (ANALM6); H89
(BNALM6);
WT161 (CNALM6); H89+WT161 (DNALM6)

Figure 4h

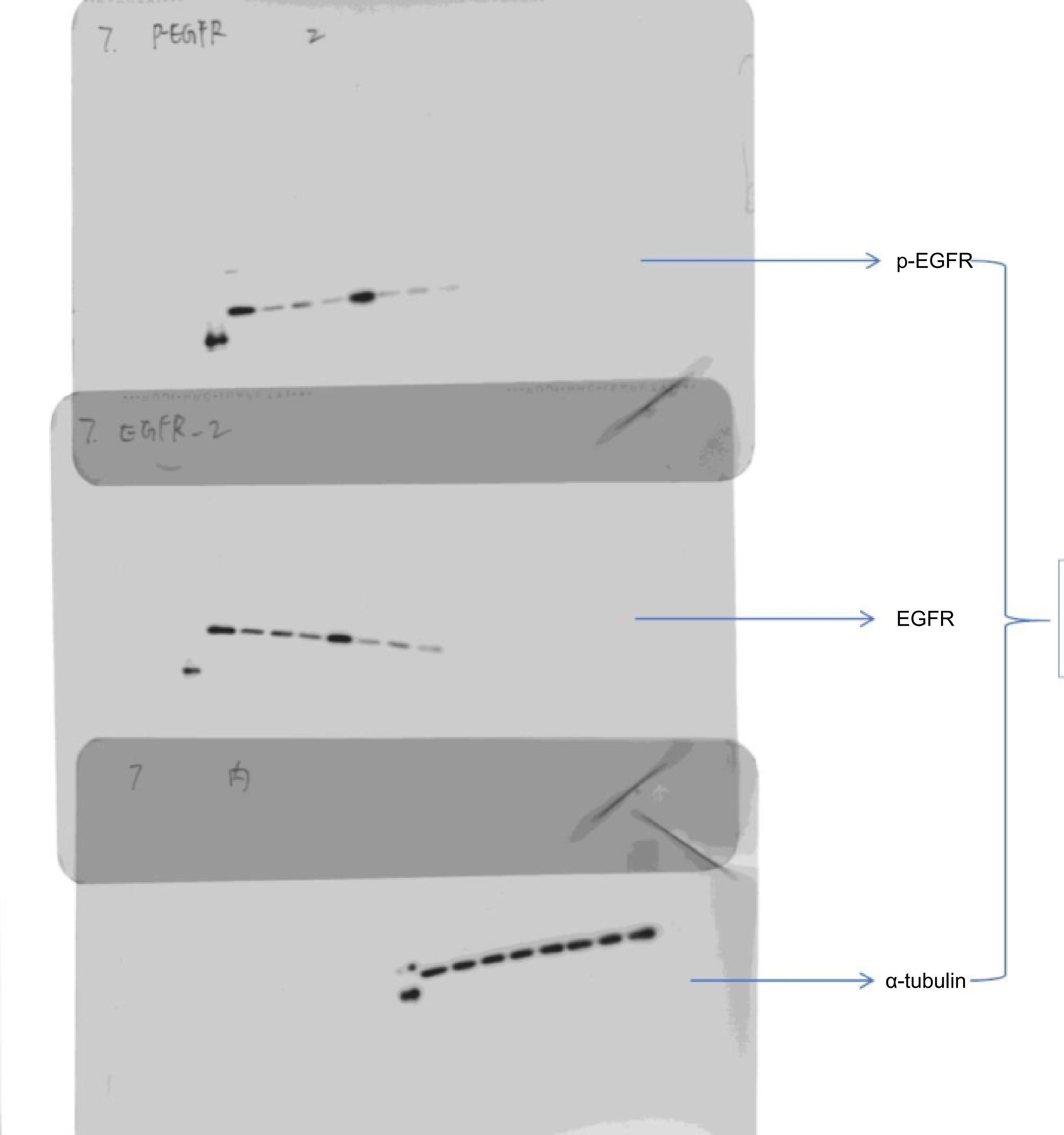

the order of the bands from left to right is:
control (AJurkat); H89 (BJurkat); WT161 (CJurkat); H89+WT161 (DJurkat); control (AMOLT-4); H89 (BMOLT-4);
WT161 (CMOLT-4); H89+WT161 (DMOLT-4)

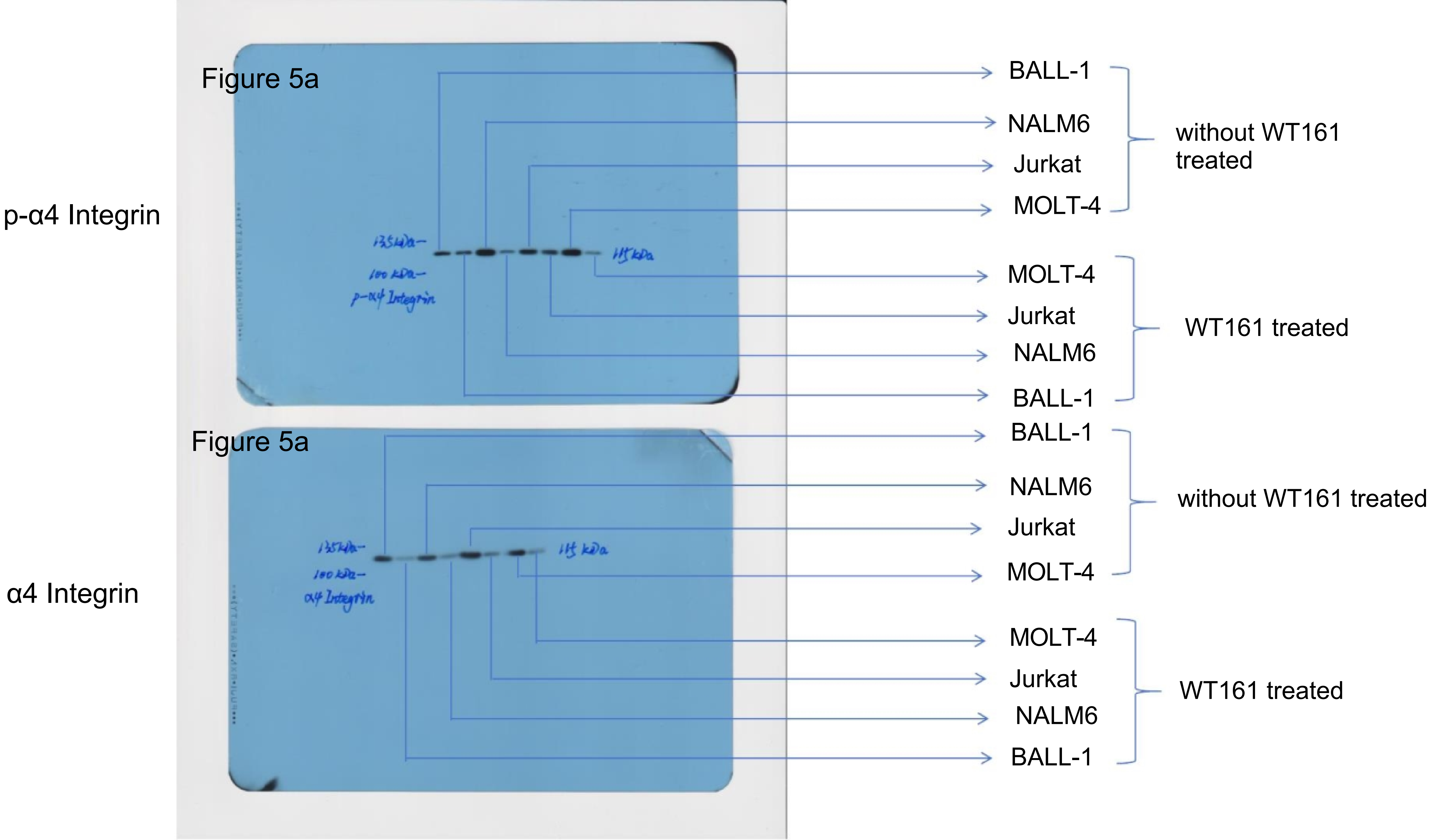
Figure 5a
p-α4 Integrin
135kDa
100 kDa
p-α4 Integrin
115 kDa
BALL-1
NALM6
Jurkat
MOLT-4
without WT161 treated
MOLT-4
Jurkat
NALM6
BALL-1
WT161 treated
Figure 5a
α4 Integrin
135kDa
100 kDa
α4 Integrin
115 kDa
BALL-1
NALM6
Jurkat
MOLT-4
without WT161 treated
MOLT-4
Jurkat
NALM6
BALL-1
WT161 treated

Figure 5a

α-tubulin

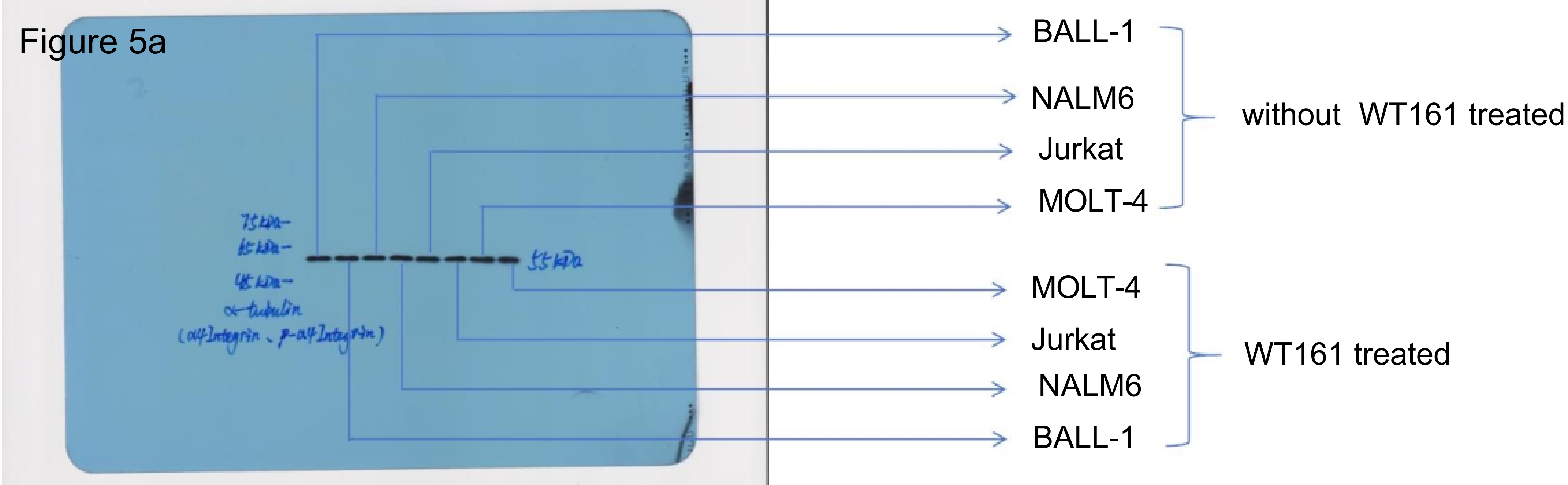

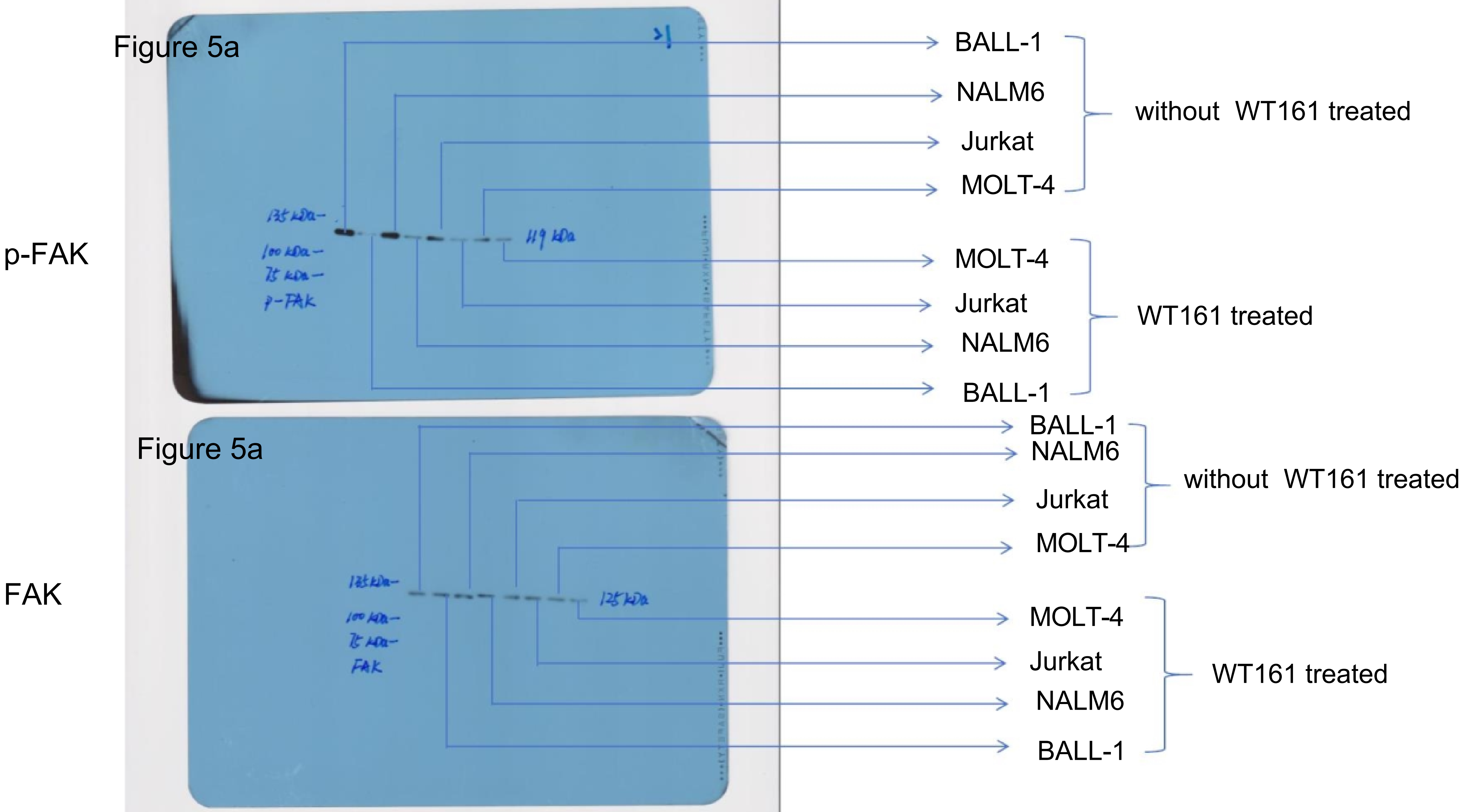
Figure 5a
p-FAK
135 kDa-
100 kDa-
75 kDa-
p-FAK
119 kDa
BALL-1
NALM6
Jurkat
MOLT-4
without WT161 treated
MOLT-4
Jurkat
NALM6
BALL-1
WT161 treated
Figure 5a
FAK
135 kDa-
100 kDa-
75 kDa-
FAK
125 kDa
BALL-1
NALM6
Jurkat
MOLT-4
without WT161 treated
MOLT-4
Jurkat
NALM6
BALL-1
WT161 treated

Figure 5a

α tubulin

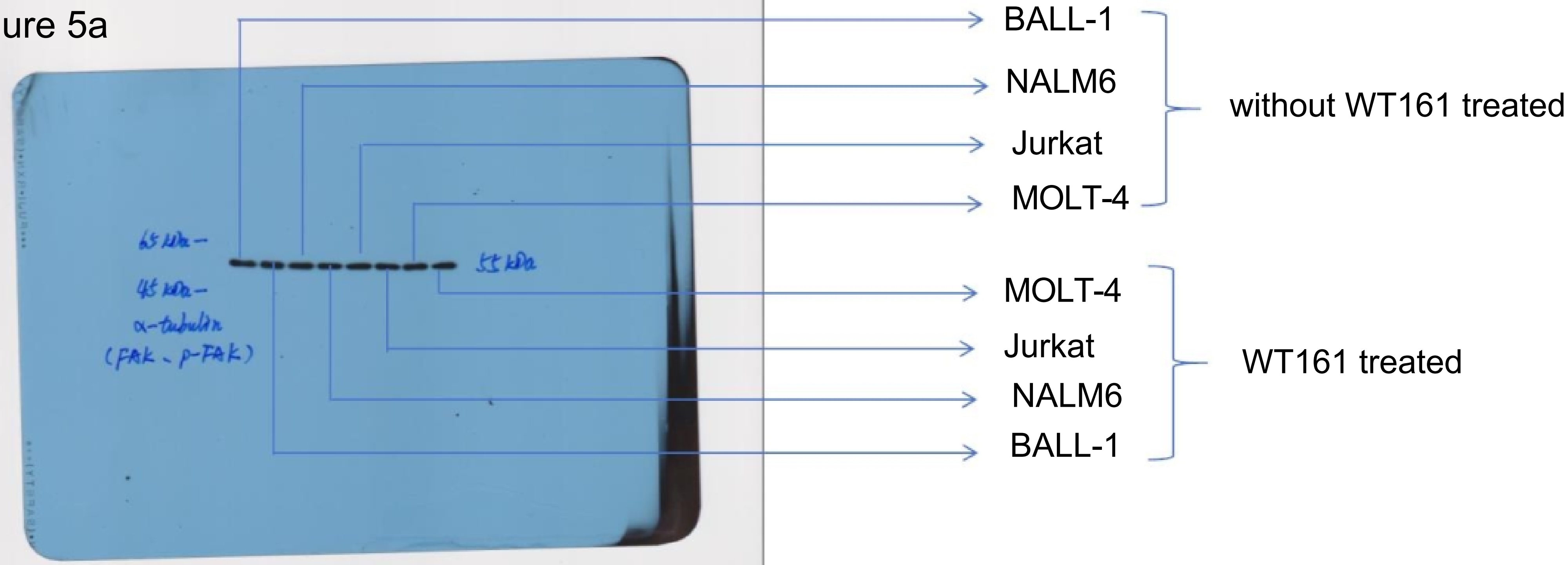

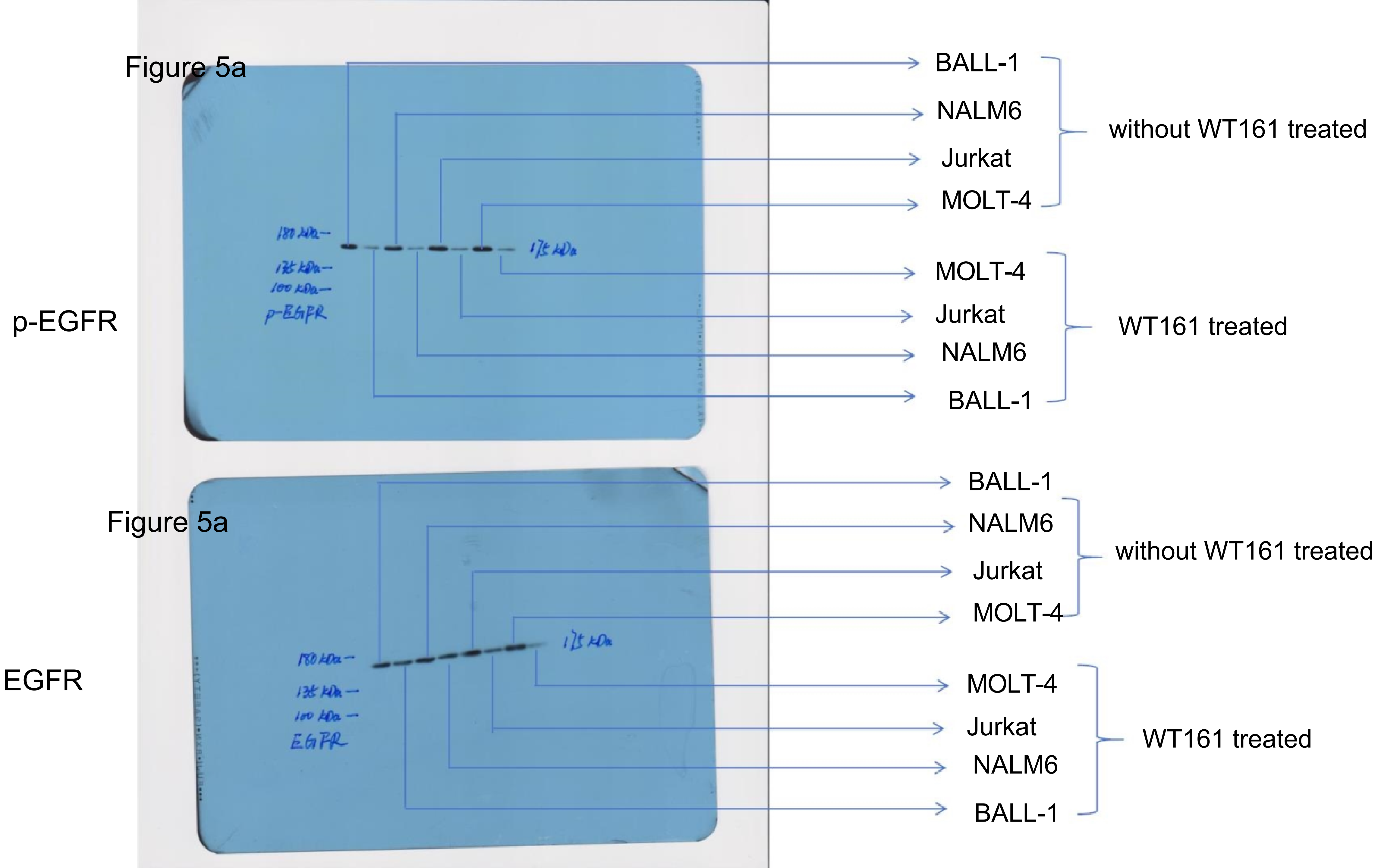
Figure 5a
p-EGFR
180 kDa
135 kDa
100 kDa
p-EGFR
175 kDa
BALL-1
NALM6
Jurkat
MOLT-4
without WT161 treated
MOLT-4
Jurkat
NALM6
BALL-1
WT161 treated
Figure 5a
EGFR
180 kDa
135 kDa
100 kDa
EGFR
175 kDa
BALL-1
NALM6
Jurkat
MOLT-4
without WT161 treated
MOLT-4
Jurkat
NALM6
BALL-1
WT161 treated

Figure 5a

α-tubulin

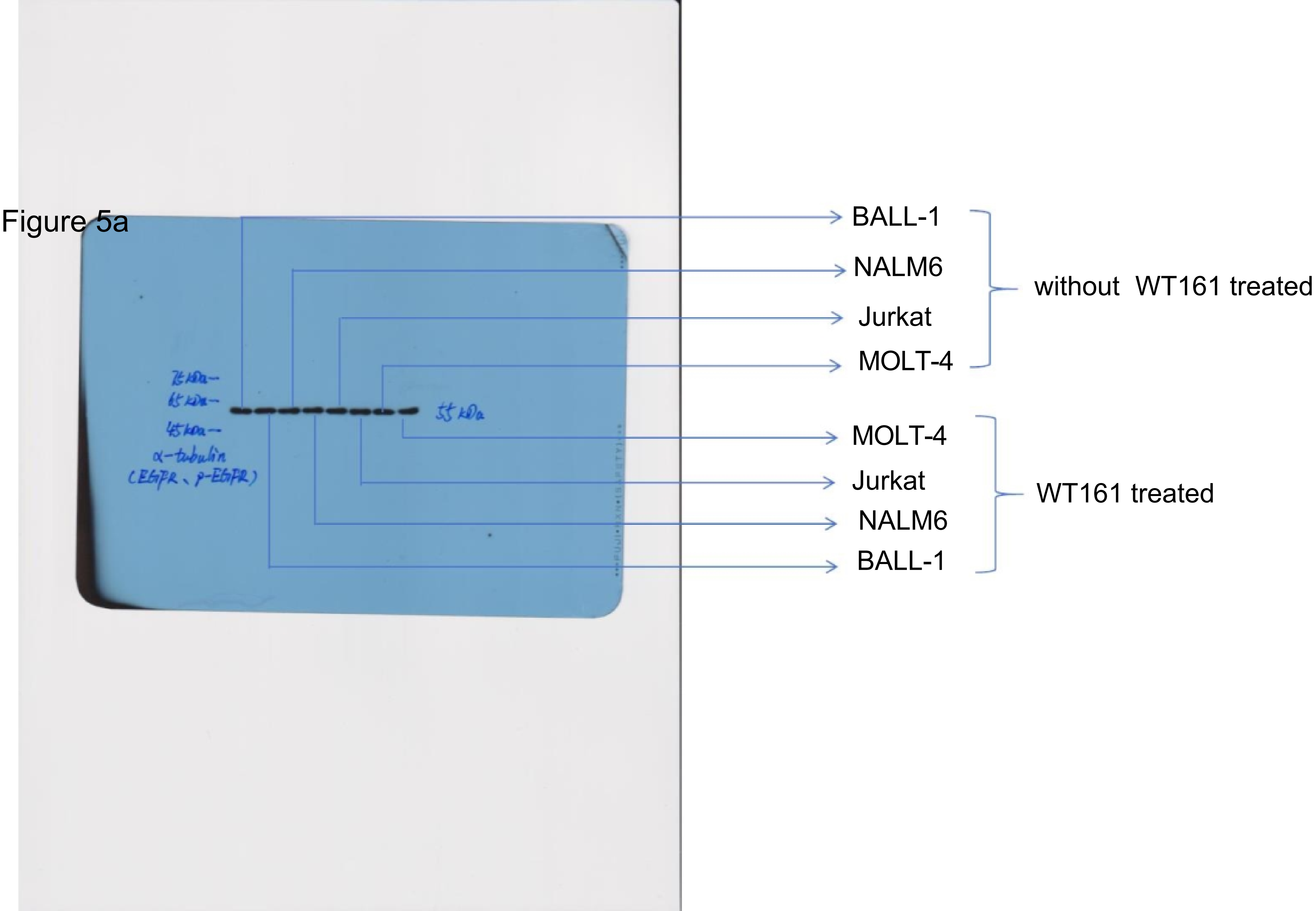

Supplement: Supplementary file 1 — Supplementary Material 1 [file 41598_2025_23887_MOESM1_ESM.pdf]
