# Supplementary figures and images for: Selective HDAC6 inhibitor WT161 modulates the VLA-4/FAK pathway by inhibiting PKA activity in acute lymphoblastic leukemia
Source: Sci Rep. 2025 Nov 17;15:40178. doi: 10.1038/s41598-025-23887-y (PMC12624111; doi:10.1038/s41598-025-23887-y)

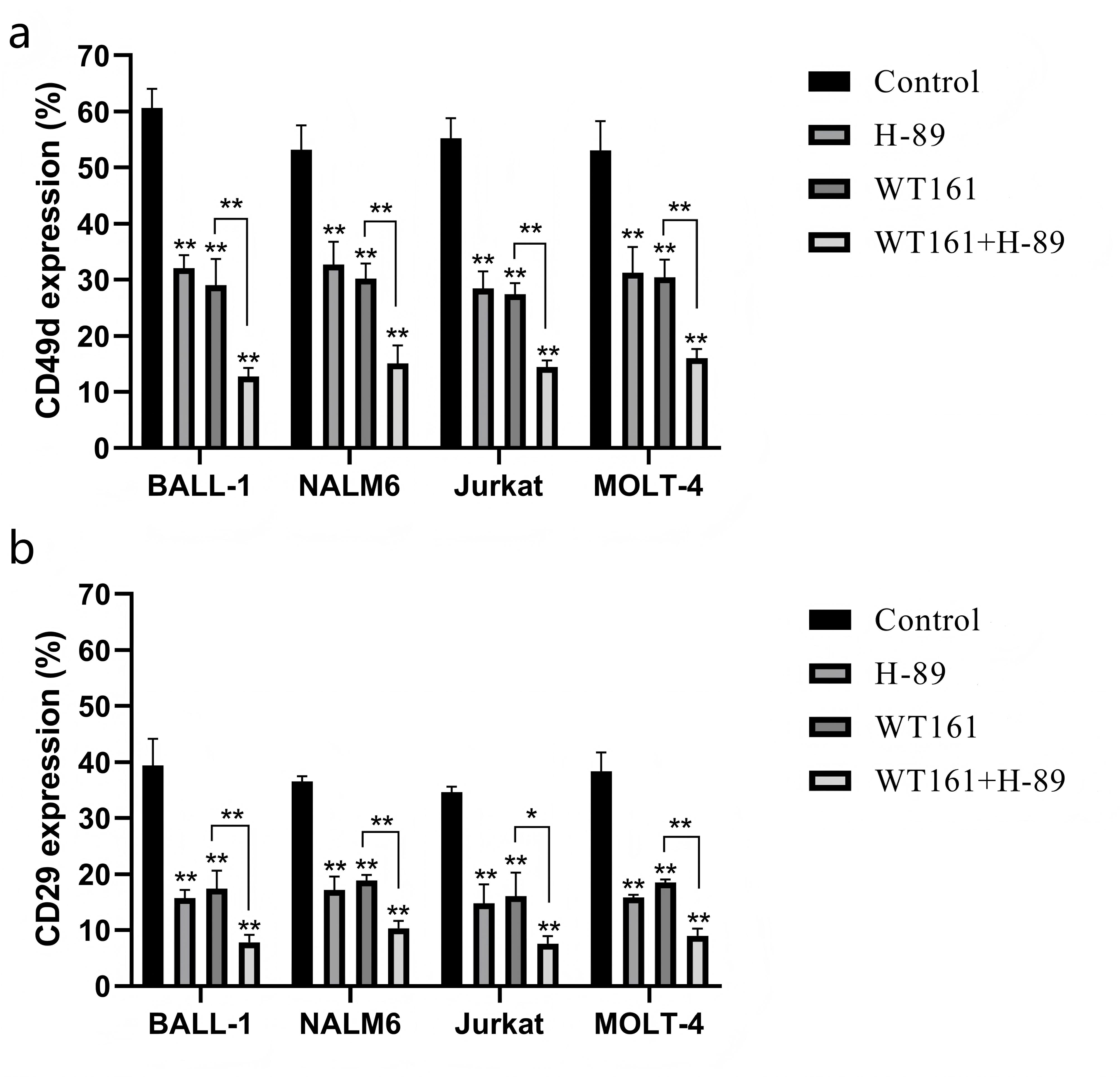

Supplement: Supplementary file 2 — Supplementary Material 2 [file 41598_2025_23887_MOESM2_ESM.jpg]
