# Supplementary material for: Selective HDAC6 inhibitor WT161 modulates the VLA-4/FAK pathway by inhibiting PKA activity in acute lymphoblastic leukemia
Source: Sci Rep. 2025 Nov 17;15:40178. doi: 10.1038/s41598-025-23887-y (PMC12624111; doi:10.1038/s41598-025-23887-y)
Supplement: Supplementary file 3 — Supplementary Material 3 [file 41598_2025_23887_MOESM3_ESM.docx]

**Figure S1: The decreased expression of CD49d/CD29 on ALL cells treated with WT161/H-89**

1. The flow cytometric analysis showing the decreased expressions of CD49d (α4-subunit）on the four ALL cell lines, including BALL-1, NALM6, Jurkat and MOLT-4 treated with WT161 and H-89. b. The flow cytometric analysis showing the decreased expressions of CD29 (β1-subunit）on the four ALL cell lines treated with WT161 and H-89. * p<0.05; ** p <0.01
